# Disruption of pathways regulated by Integrator complex in Galloway–Mowat syndrome due to *WDR73* mutations

**DOI:** 10.1038/s41598-021-84472-7

**Published:** 2021-03-08

**Authors:** F. C. Tilley, C. Arrondel, C. Chhuon, M. Boisson, N. Cagnard, M. Parisot, G. Menara, N. Lefort, I. C. Guerrera, C. Bole-Feysot, A. Benmerah, C. Antignac, G. Mollet

**Affiliations:** 1 Laboratory of Hereditary Kidney Diseases, Imagine Institute, INSERM UMR 1163, Université de Paris, 75015 Paris, France; 2 Proteomics Platform 3P5-Necker, Structure Fédérative de Recherche Necker, INSERM US24/CNRS UMS 3633, Université de Paris, 75015 Paris, France; 3 Bioinformatic Core Facility, Structure Fédérative de Recherche Necker, INSERM US24/CNRS UMS 3633, Imagine Institute, Université de Paris, 75015 Paris, France; 4 Genomics Core Facility, Imagine Institute, Structure Fédérative de Recherche Necker, INSERM U1163 and INSERM US24/CNRS UMS3633, Université de Paris, 75015 Paris, France; 5 iPSC Core Facility, Imagine Institute, INSERM UMR 1163, Université de Paris, 75015 Paris, France; 6 Département de Génétique, AP-HP, Hôpital Necker-Enfants Malades, 75015 Paris, France

**Keywords:** Immunochemistry, RNA, Gene expression, Gene regulation, Neurodevelopmental disorders, RNA splicing, Cell division, Mechanisms of disease

## Abstract

Several studies have reported *WDR73* mutations to be causative of Galloway–Mowat syndrome, a rare disorder characterised by the association of neurological defects and renal-glomerular disease. In this study, we demonstrate interaction of WDR73 with the INTS9 and INTS11 components of Integrator, a large multiprotein complex with various roles in RNA metabolism and transcriptional control. We implicate WDR73 in two Integrator-regulated cellular pathways; namely, the processing of uridylate-rich small nuclear RNAs (UsnRNA), and mediating the transcriptional response to epidermal growth factor stimulation. We also show that WDR73 suppression leads to altered expression of genes encoding cell cycle regulatory proteins. Altogether, our results suggest that a range of cellular pathways are perturbed by WDR73 loss-of-function, and support the consensus that proper regulation of UsnRNA maturation, transcription initiation and cell cycle control are all critical in maintaining the health of post-mitotic cells such as glomerular podocytes and neurons, and preventing degenerative disease.

## Introduction

First described in 1968, Galloway–Mowat syndrome (GAMOS) is an extremely rare condition characterised by the co-occurrence of various neurological symptoms and glomerular-renal disease^1^. Neurological symptoms include either primary or post-natal microcephaly, often with brain anomalies such as cerebral atrophy/hypoplasia and neural migration defects^2^. Renal manifestations range from proteinuria to steroid-resistant nephrotic syndrome (SRNS), which rapidly progresses to end-stage renal disease^3,4^. Hereditary SRNS generally occurs following mutation of genes encoding proteins required for podocyte functions; podocytes being specialised epithelial cells which form part of the glomerular filtration barrier^5^. Both neurons and podocytes are highly differentiated post-mitotic cells, and share several morphological similarities, including an elaborate cytoskeleton and specialised cell–cell junctions^6^. Based on their morphological and biochemical similarities, it is reasonable to suppose that neurons and podocytes are sensitive to perturbations in the same cellular pathways.

The first gene to be implicated in GAMOS pathogenesis was *WDR73*, with two loss-of-function mutations identified in two families in 2014^3^. Since this time, a number of other pathogenic mutations in *WDR73*, including some missense mutations, have been reported by several research groups^4,7–11^. GAMOS patients with *WDR73* mutations are affected by a particular subset of the disorder, typically presenting with post-natal progressive microcephaly, ataxia with cerebellar degeneration, and sometimes epilepsy and optic atrophy^8^. Renal disease in these patients tends to be of later and more variable age of onset compared to patients with mutations in genes encoding, for example, the KEOPS complex^12^.

Ironically, despite being the first gene implicated in GAMOS, *WDR73* encodes a protein perhaps the least well-characterised amongst all those linked to the disease. Roles for WDR73 in the regulation of microtubule dynamics, cell cycle progression, mTOR signalling and pyrimidine base synthesis have been proposed^3,4^. In a zebrafish model, *WDR73* knockdown by morpholino is linked to disrupted brain morphogenesis, perturbed neuronal differentiation and a reduction in the number of mitotic neural progenitor cells (NPCs)^9^. Despite these advances, the precise cellular function of WDR73, and thus how exactly mutations in *WDR73* lead to GAMOS development, remains poorly understood.

In this study, we use a combination of proteomic, biochemical and RNA analyses to further elucidate the function of WDR73. Firstly, we describe and characterise an interaction between WDR73 and the proteins which make up the catalytic subunit of the Integrator complex, Integrator complex subunit 9 (INTS9) and Integrator complex subunit 11 (INTS11). Integrator is a large RNA polymerase II (RNAPII)-binding complex possessing various roles in transcriptional control and RNA metabolism, including the processing of uridylate-rich small nuclear RNAs (UsnRNAs) and promoting RNAP-II pause-release downstream of growth factor stimulation^13,14^. Secondly, we report deregulation of these two Integrator-regulated pathways in cells in which *WDR73* is either mutated or its expression suppressed by siRNA. Finally, we show that WDR73-depletion in podocytes correlates with altered expression of several genes encoding cell cycle regulatory proteins. Taken together, our results underscore an emerging consensus that perturbed cell cycle control may contribute to the mechanism of pathogenesis in patients with *WDR73*-linked GAMOS^4,9^.

## Results

### WDR73 interacts with Integrator complex components INTS9 and INTS11

**Figure 1:**
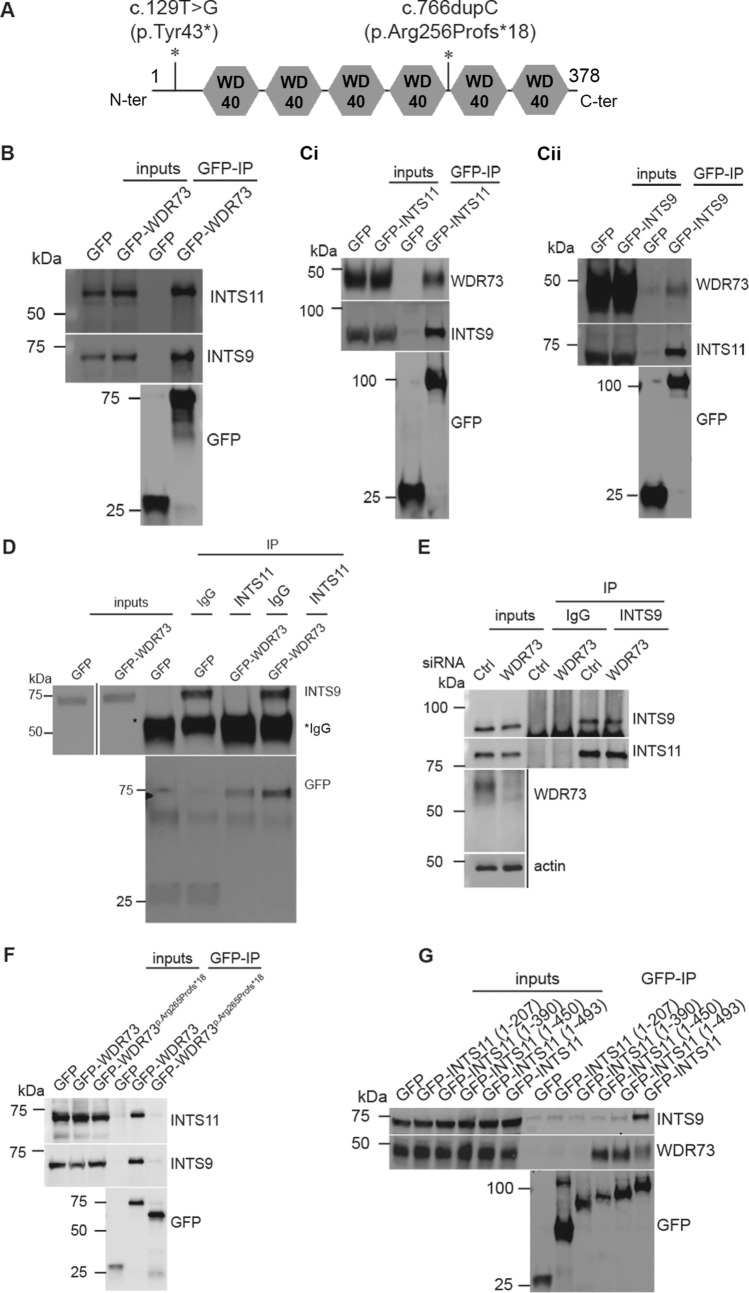
WDR73 interacts with Integrator complex components INTS9 and INTS11. (**A**) Schematic representation of WDR73 protein with its six predicted WD40 repeats (grey hexagons) and the position of GAMOS-associated mutations described in this article (asterisk). (**B**) GFP-IP from immortalised podocytes stably expressing either GFP or GFP-WDR73 shows interaction of GFP-WDR73 with endogenous INTS9 and INTS11. (**Ci**,**ii**) GFP-IP from HEK293T cells transiently transfected with either GFP-INTS11 (**i**) or GFP-INTS9 (**ii**) shows interaction of both tagged proteins with endogenous WDR73. (**D**) Immunoprecipitation of INTS11 from podocytes stably expressing either GFP or GFP-WDR73 shows that endogenous INTS11 may associate with GFP-WDR73. (**E**) Immunoprecipitation of endogenous INTS9 from control siRNA treated or WDR73-suppressed podocytes shows that WDR73 suppression has no effect on the ability of INTS9 and INTS11 to associate. Please note WDR73 and actin bands are cropped for clarity, as WDR73 lane shows only contamination from IgG in IP lanes. (**F**) GFP-IP from HEK293T cells transiently transfected with 2 µg DNA encoding GFP-WDR73, or 10 µg DNA encoding either GFP only or GFP-WDR73 p.256Profs*18 shows that the latter construct is unable to associate with either INTS9 or INTS11. (**G**) GFP-IP from HEK293T cells transiently transfected with the indicated constructs shows that WDR73 is able to associate with GFP-INTS11 wild type, GFP-INTS11 (1-450) and (1-493). All experiments shown are representative of at least three independent biological repeats.

WDR73 is a protein predicted to contain six WD40 repeats, these latter being known to mediate diverse protein–protein or protein-DNA interactions (Fig. 1A).To further understand in which cellular pathways WDR73 participates in, we first performed a proteomic analysis of GFP immunoprecipitates isolated from a conditionally immortalised human podocyte cell line stably expressing GFP-WDR73 full length (Supplementary Fig. S1). Among the 29 proteins identified as putative WDR73-interacting proteins (Supplementary Table S1), the two most significantly enriched proteins in the GFP-WDR73 were Integrator complex subunit 9 (INTS9) and Integrator complex subunit 11 [INTS11, otherwise known as cleavage and polyadenylation specificity factor-3 like (CPSF3L)]. Most interestingly, mutations in genes encoding the Integrator subunits INTS1 and INTS8 have been linked to a neurological disorder strikingly reminiscent of that affecting GAMOS patients with *WDR73* mutations^15^.

We were able to confirm the interaction between GFP-WDR73 and endogenous INTS9 and INTS11 in podocytes (Fig. 1B). In the reverse direction, we were able to immunoprecipitate endogenous WDR73 with both GFP-INTS11 and GFP-INTS9 transiently transfected into HEK293T cells; with more WDR73 consistently immunoprecipitated by INTS11 (Fig. 1Ci,ii). We then demonstrated immunoprecipitation (IP) of GFP-WDR73 from podocytes stably overexpressing this construct using an antibody against endogenous INTS11 (Fig. 1D).

Integrator is reported to be a nuclear complex^16^. We found expression of endogenous WDR73 in both nuclear and cytoplasmic extracts isolated from HeLa cells (Supplementary Fig. S1A), confirming that WDR73 may localise to the same cellular compartments as Integrator. In addition, GFP-WDR73 stably expressed in podocytes showed a diffuse staining in both compartments (Supplementary Fig. S2B). However, due to concerns regarding antibody specificity, we were unable to investigate subcellular localisation of endogenous WDR73 protein by immunofluorescence, and concomitantly whether WDR73 colocalises with endogenous INTS9 and INTS11 in imaging experiments (Supplementary Fig. S2C).

We also assessed whether WDR73 was capable of interacting with other members of the Integrator complex. We chose to test WDR73 association with INTS4, a protein reported to directly bind and scaffold the INTS9-INTS11 heterodimer^17^. As expected, we were able to demonstrate immunoprecipitation of INTS4 by GFP-INTS11, yet found no association between INTS4 and GFP-WDR73 (Supplementary Fig. S3A). We then investigated whether WDR73 functions to regulate the INTS9-INTS11 interaction. To this end, we suppressed WDR73 using siRNA in podocytes and then assessed the ability of INTS9 to immunoprecipitate INTS11. We found that depletion of WDR73 had no effect on the ability of INTS9 to immunoprecipitate INTS11 (Fig. 1E, Supplementary Fig. S3B).

In order to determine whether a GAMOS-associated *WDR73* mutation affected the ability of WDR73-INTS9/INTS11 interaction, we introduced the c.766dup.C mutation, encoding the variant p.Arg256Profs*18, into GFP-WDR73 (Fig. 1A). We were able to demonstrate that WDR73 p.Arg256Profs*18 could not associate with both INTS9 and INTS11 (Fig. 1F). As the p.Arg256Profs*18 mutant variant lacks a proportion of the WDR73 C-terminal domain containing two WD40 repeats, we hypothesised that the WDR73-INTS9/INTS11 interaction was mediated by this region. However, using two truncated GFP-WDR73 constructs, one constituting the N-terminal region (1-256), and the other the C-terminal region (257-378), we found neither construct to be capable of interaction with endogenous INTS9 or INTS11 (Supplementary Fig. S3C). This suggests that only full length WDR73 protein is capable of interaction with Integrator, an unsurprising result, as loss of any of its WD40-repeats would likely lead to misfolding of the whole structure.

Since GFP-INTS11 seems to consistently immunoprecipitate more endogenous WDR73 than GFP-INTS9 (Fig. 1Ci,ii), we hypothesised that WDR73 was perhaps interacting with the INTS9-INTS11 heterodimer via direct association with INTS11. Using the INTS11 domain boundaries outlined in a recent article^18^, we subcloned various fragments of INTS11 into a GFP-expression vector, and then tested them for WDR73 and INTS9 association (Fig. 1G). As previously reported, we showed that the GFP-INTS11 (1-493) construct, which lacks the C-terminal amino acids 499-600, was unable to bind INTS9. In contrast, we found that in addition to GFP-INST11 wild-type, both GFP-INTS11 (1-450) and GFP-INTS11 (1-493) were able to immunoprecipitate WDR73. GFP-INTS11 (1-390) exhibited no interaction, suggesting that WDR73 associates with INTS11 between residues 390 and 450.

If WDR73 is also able to associate with INTS9, we might expect that an INTS11 mutant incapable of INTS9 association would immunoprecipitate less WDR73. To test this, we introduced a mutation encoding an p.Leu509Ala amino acid substitution into GFP-INTS11 (GFP-INTS11^p.L509A^); this mutation having previously been shown to cause disassociation of the INTS9-INTS11 heterodimer^18^. As expected, we observed no association of GFP-INTS11^p.L509A^ with INTS9, and also found a trend for less WDR73 to be immunoprecipitated by this construct compared to wild-type INTS11 (Supplementary Fig. S3Di,ii). However, this result contrasted with the observation in Fig. 1D that INTS9 is not immunoprecipitated with INTS11 constructs (1-450 and 1-493) capable of association with WDR73 (Fig. 1G). Altogether, our results confirm that WDR73 is able to associate with the INTS11/INTS9 complex, probably directly with INTS11.

### GAMOS patient-derived NPCs contain altered levels of unprocessed UsnRNAs

**Figure 2:**
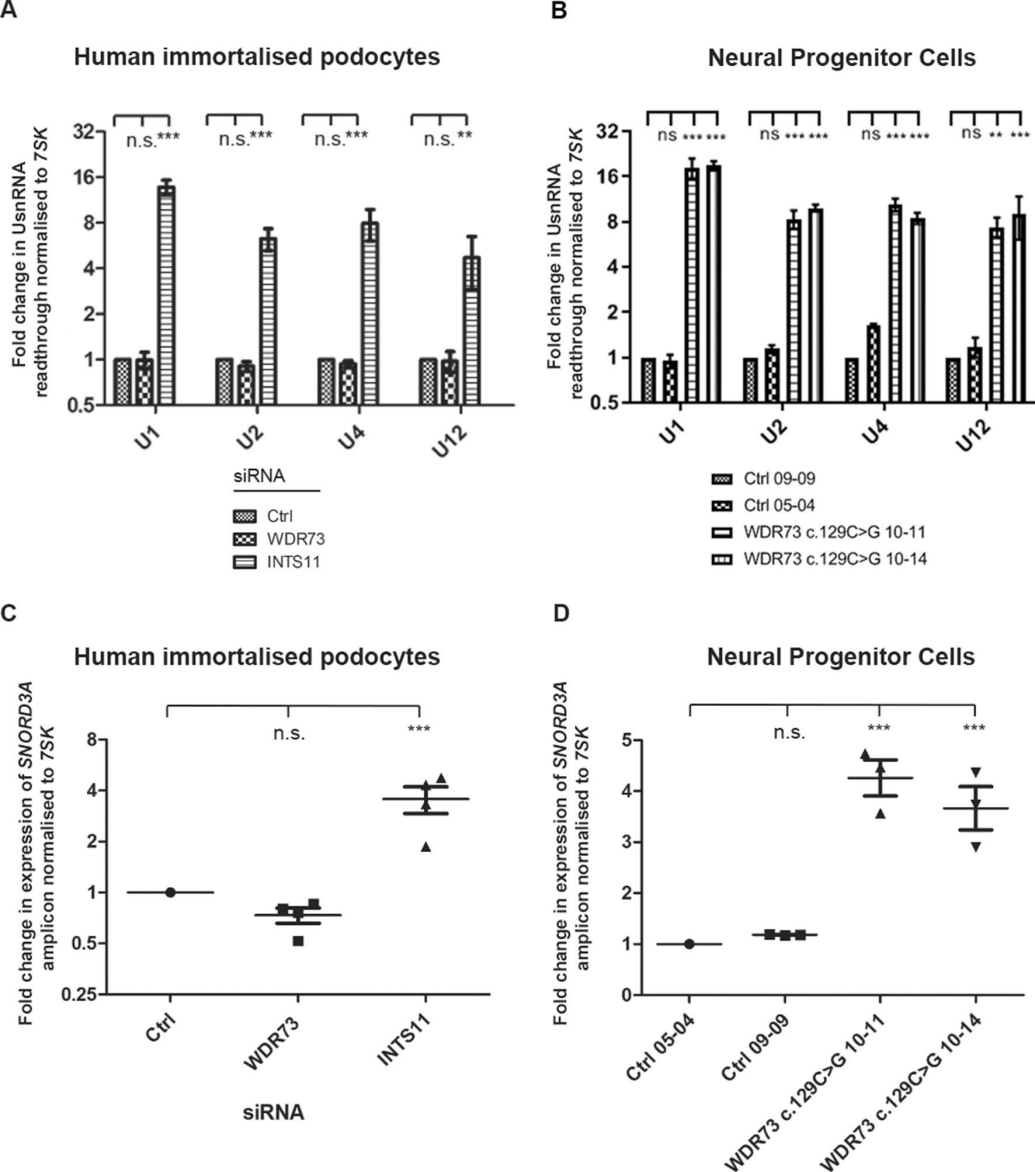
GAMOS patient-derived NPCs contain altered levels of unprocessed UsnRNAs. (**A**,**B**) RT-qPCR analysis showing expression of unprocessed U1, U2, U4 and U12 snRNA transcripts in control siRNA-treated or WDR73 and INTS11 suppressed immortalised podocytes (**A**), and in two NPC clones derived from a patient with WDR73-linked GAMOS compared to NPCs derived from two healthy controls (**B**). (**C**,**D**) qRT-PCR showing expression levels of long *SNORD3A* amplicon in either control siRNA-treated and WDR73 or INTS11 –suppressed podocytes (**C**) and in from a patient with WDR73-linked GAMOS compared to NPCs derived from two healthy controls (**D**). With the exception of the experiment assessing *U12* levels in NPCs, in which only two control RNA samples were available, (A and C) each show data from three independent biological repeats. Error bars are ± S.E.M.; n.s indicates difference between conditions is not significant, and *, ** and *** that *p* < 0.05, 0.01 and 0.001 respectively as determined by a one-sample t-test.

We then sought to determine whether WDR73 functions to regulate the same cellular processes as the Integrator complex. The first and most well-described role of the Integrator complex is in mediating the 3′ co-transcriptional cleavage of UsnRNAs, which form the nucleic acid component of the spliceosome^16,19^. We found that although siRNA-mediated suppression of INTS11 in a podocyte cell line led to a large increase in levels of primary transcripts encoding the major spliceosomal UsnRNAs U1, U2 and U4, and the minor spliceosome component U12, WDR73 knockdown had no effect (Fig. 2A, Supplementary Fig. S4).

We hypothesised that perhaps the requirement for WDR73 in UsnRNA processing was cell type dependent, and therefore compared levels of unprocessed mRNA transcripts encoding U1, U2, U4 and U12 in two NPC lines derived from induced pluripotent stem cells (iPSCs) obtained from a GAMOS patient with the c.129C > G *WDR73* mutation and in two unrelated control NPC lines. The c.129C > G substitution, encodes a premature stop codon at residue 43 (p.Tyr43*) and is considered a total loss-of-function mutation. We found significantly increased levels of unprocessed transcripts for U1, U2 and U4 snRNAs, and a trend for levels of unprocessed transcripts encoding U12 transcripts to be increased in the GAMOS patient-derived NPCs compared to control (Fig. 2B).

It has recently been proposed that Integrator may mediate the 3′ cleavage of small nucleolar RNAs (snoRNAs), a type of small RNA which function to guide chemical modifications on other RNA species^20^. We therefore analysed levels of unprocessed *SNORD3A* transcripts, choosing to analyse them since, unlike the vast majority of snoRNA genes, *SNORD3A* is not located within an intron of another gene and is instead independently transcribed^21^. Similar to our results for major and minor UsnRNAs, we observed increased expression of a long *SNORD3A* amplicon in both INTS11-suppressed podocytes and in NPCs derived from the patient with *WDR73*-linked GAMOS relative to control (Fig. 2C,D). This finding potentially implicates INTS11 and WDR73 in snoRNA processing.

### WDR73-depleted immortalised podocytes have reduced capacity to respond to EGF-stimulation

Another role ascribed to the Integrator complex is to mediate the transcriptional response to growth factor stimulation. Indeed, it has been shown that suppression of either Integrator component INTS1 or INTS11 leads to reduced activation of epidermal growth factor (EGF)-early genes following addition of EGF^22,23^.

**Figure 3:**
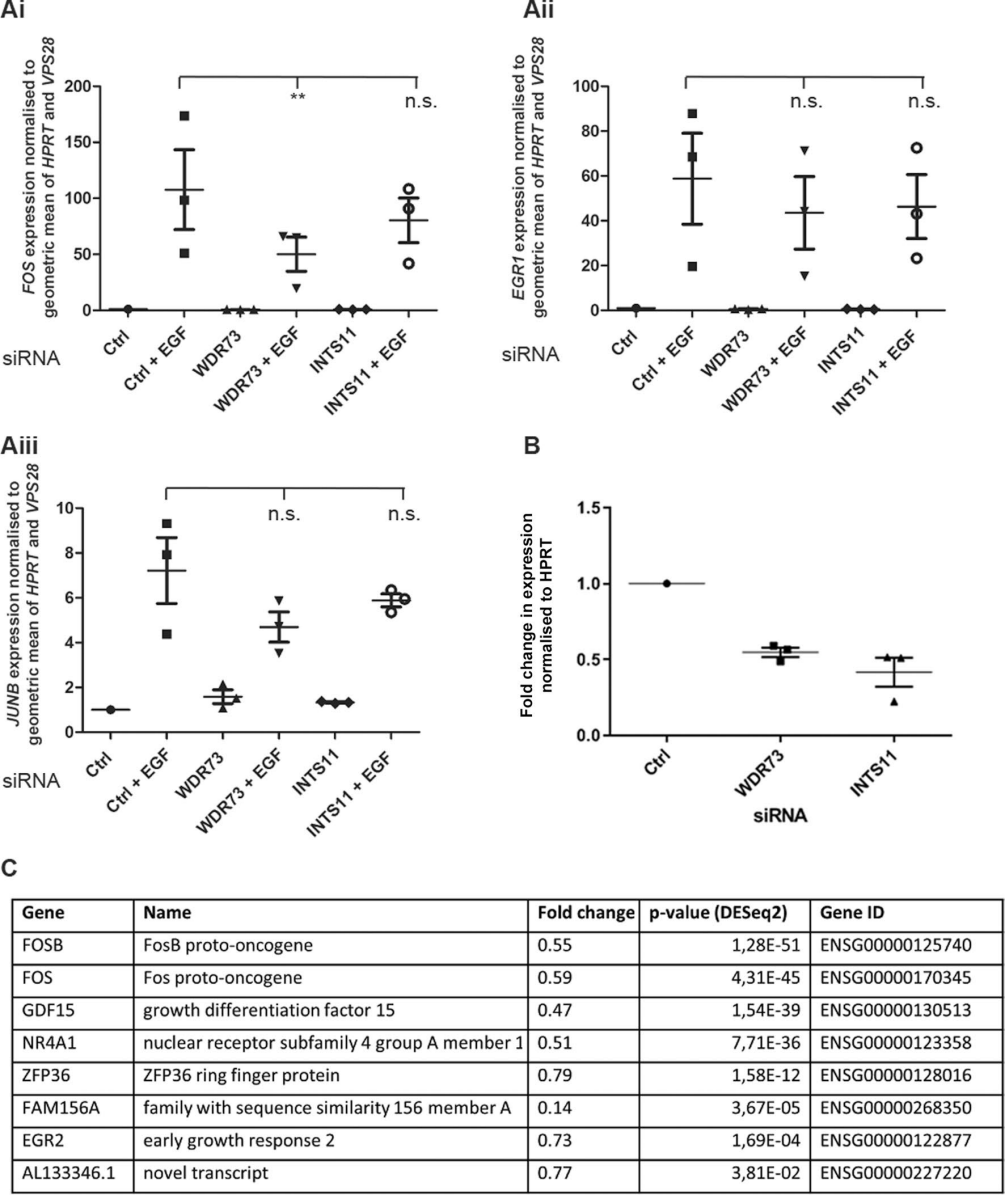
WDR73-depleted immortalised podocytes have reduced capacity to respond to EGF-stimulation. (**Ai–iii**) RT-qPCR showing expression levels of *FOS* (**Ai**), *EGR1* (**Aii**) and *JUNB* (**Aiii**) in WDR73 or INTS11 suppressed cells both before and after 30 min of EGF-stimulation compared to control. Graphs represent data from three independent experiments. Error bars are ± S.E.M.; n.s indicates difference between conditions is not significant, and *, ** and *** that *p* < 0.05, 0.01 and 0.001 respectively as determined by a one-way ANOVA test performed on normalised ΔCt values. (**B**) RT-qPCR analysis showing efficiency of *WDR73* and *INTS11* knockdown in experiments presented in (**A**,**C**) RNA-sequencing was performed on RNA extracted from control siRNA-treated and WDR73 suppressed human podocytes, either with or without 30minutes EGF stimulation. Genes shown are those which were both induced at least twofold in the Ctrl + EGF condition compared to Ctrl − EGF, and whose levels were also reduced in the WDR73 + EGF condition compared to Ctrl + EGF. Data shown are representative of three technical repeats. For clarity, only the *p* value from the DESeq2 analysis is shown in the table.

We wanted to investigate whether WDR73 also played a role in promoting the transcription of EGF-early genes downstream of EGF stimulation. We therefore suppressed WDR73 using siRNA in human immortalised podocytes and subjected the cells to serum starvation for 24 h before stimulating them with 100 ng/ml EGF for 30 min. We then used RT-qPCR to compare induction of a selection of EGF-early genes in INTS11- and WDR73-depleted podocytes to control siRNA-transfected cells. Based on reports implicating INTS11 in their transcriptional induction, we chose to first test induction of *FOS* and *EGR1*^22,23^, and also included the EGF-early gene *JUNB* (*AP-1*)^24^ (Fig. 3Ai–iii). Expression of *FOS*, *EGR1* and *JUNB* mRNA were induced following EGF stimulation in both control and WDR73 and INTS11-depleted cells. However, we found that *FOS* levels were consistently and significantly lower in WDR73-depleted podocytes following 30 min of EGF stimulation compared to control. *JUNB* and *EGR1* also showed a trend to be upregulated to a lesser extent compared to control following WDR73 knockdown, although this effect did not reach statistical significance. Importantly, we demonstrated by western blot that WDR73 depletion did not affect levels of the EGF-receptor (Supplementary Fig. S5Ai–ii). We included INTS11-depleted cells in this experiment, however, despite robust knockdown of *INTS11* at the mRNA level (Fig. 3B), we observed no consistent and significant effect of INTS11 depletion on induction of either *FOS*, *EGR1* or *JUNB*.

To investigate whether WDR73-suppression affected the induction of any other EGF-early genes, we performed RNA-sequencing (RNA-Seq) on control siRNA-treated and WDR73-depleted podocytes, either with or without EGF stimulation (Ctrl +/− EGF and WDR73 KD +/− EGF). We identified 1694 genes that had significantly reduced expression in the WDR73 KD + EGF condition compared to Ctrl + EGF (Supplementary Tables S2 and S3). In order to identify genes whose expression change could be due to reduced transcriptional induction following EGF stimulation, rather than simply due to the effect of WDR73 knockdown, we cross-referenced the Ctrl + EGF vs WDR73 KD + EGF dataset with the dataset of genes exhibiting at least a twofold increase in expression between the Ctrl + EGF vs Ctrl − EGF condition (Supplementary Tables S4and S5). The genes in this dataset that were induced to a lesser extent in WDR73 KD + EGF compared to Ctrl + EGF are shown in Fig. 3C. We were able to confirm our finding that WDR73-depletion results in reduced activation of *FOS* following EGF-stimulation, and also observed reduced induction of the classical EGF-early genes *EGR2*, *FOSB* and *NR4A1*. Other genes apparently induced by EGF stimulation which were expressed to a lower extent in the WDR73 KD + EGF compared to control included *ZFP36*, *FAM156A*, *SNX32* and *GDF15*, encoding tristetraprolin, FAM156A (a protein of largely undescribed function), the trafficking protein sorting-nexin 32 and the macrophage inhibitory molecule growth/differentiation factor 15. From this experiment, we can conclude that WDR73 depletion is linked to reduced induction of a subset of EGF-early genes. However, taking a comprehensive view of our RNA-Seq results, we can see that, rather than causing global transcriptional downregulation, WDR73 suppression in both unstimulated and EGF-stimulated conditions is also linked to upregulation of certain genes (Supplementary Fig. S5Bi,ii).

### WDR73 and INTS11 suppression is linked to altered expression of cell cycle regulatory genes

The ability of a cell to progress through the cell cycle is dependent on its ability to respond to growth factor stimulation^25^. Previous work characterising the cellular function of WDR73 has described defective cell proliferation in cells harbouring *WDR73* mutations^4,9^. We therefore might expect that the physiological function of WDR73 is to promote cell division and mitosis, and indeed, our results showing reduced induction of EGF-early genes in WDR73 knockdown podocytes following EGF-stimulation are consistent with this hypothesis. Ingenuity pathway analysis of the list of genes differentially expressed between Ctrl -EGF and WDR73 KD -EGF conditions, revealed that the category of functions the most significantly enriched in the WDR73 KD -EGF condition was cancer (Supplementary Fig. S6A). A famous hallmark of cancer is deregulated cell cycle control^26^, and indeed, we found altered expression of genes encoding many cyclin and cyclin-regulatory proteins in our WDR73 KD -EGF dataset compared to control.

However, in comparing the list of genes differentially expressed between the Ctrl -EGF and WDR73 KD -EGF conditions (i.e. steady state) (Supplementary Tables S6 and S7), we noticed that the gene most significantly upregulated upon WDR73 knockdown was *CCND1*, encoding the protein cyclin D1. The most well described role of cyclin D1 is to drive G1/S cell cycle phase transition^27^.

**Figure 4:**
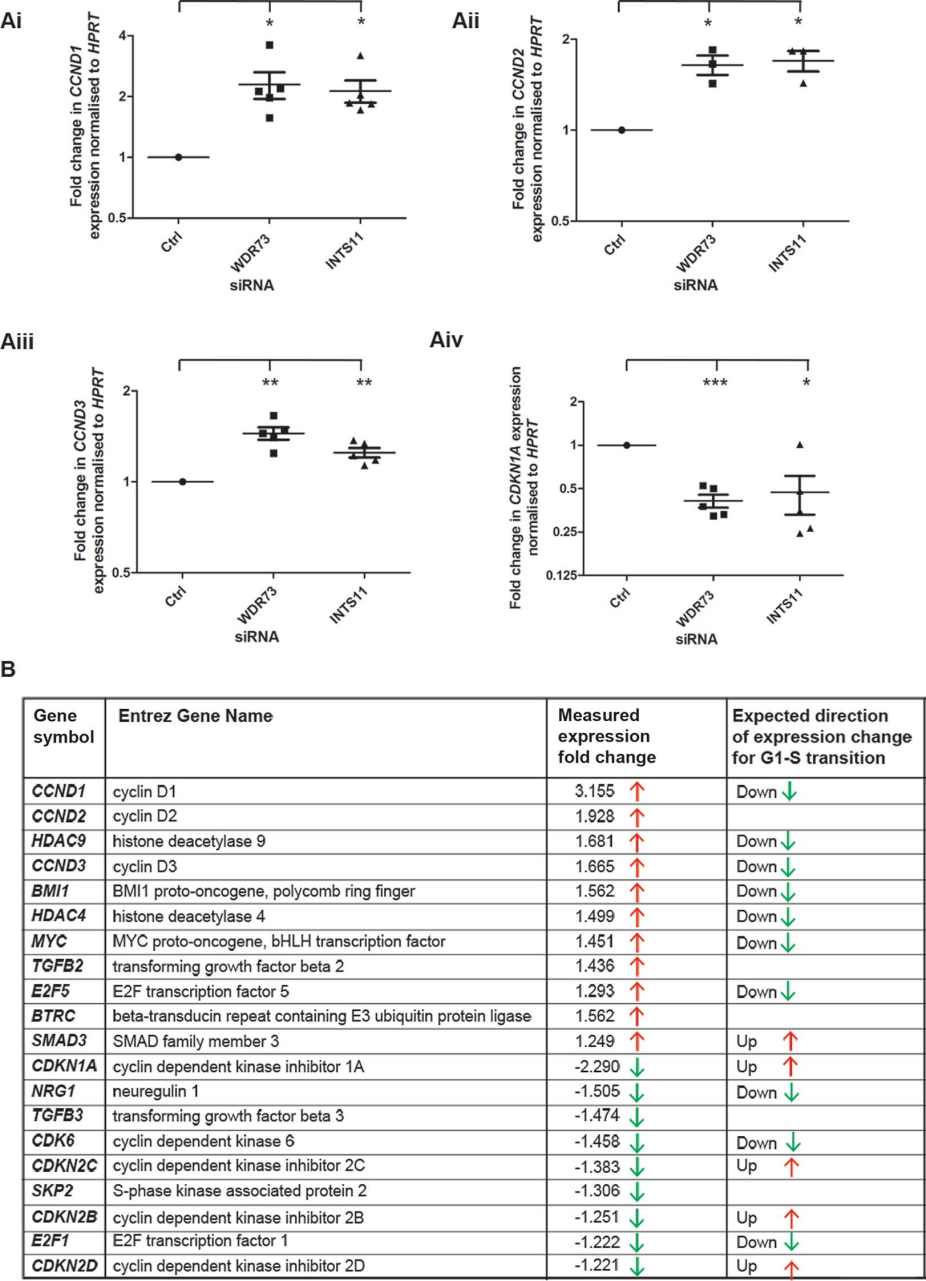
WDR73 and INTS11 suppression is linked to altered expression of cell cycle regulatory genes. (**Ai–iv**) RT-qPCR showing expression levels of *CCND1*, *CCND2*, *CCND3* and *CDKN1A* in control siRNA treated and WDR73 and INTS11 suppressed human podocytes. Graphs show data from at least three independent experiments. Error bars are ± S.E.M.; *, ** and *** indicate that *p* < 0.05, 0.01 and 0.001 respectively as determined by a one-sample t-test. (**B**) Ingenuity pathway analysis of significantly differentially expressed genes in WDR73 siRNA-treated cells − EGF compared to control siRNA-treated cells − EGF reveals that WDR73 knockdown results in altered expression of genes which promote G1-S cell cycle progression. ‘Expected’ column with green and red arrows shows the direction of gene expression change which is expected to participate in the activation of the G1-S transition. ‘Measured experimental fold change’ column shows average observed fold change in expression following WDR73 knockdown and the direction of gene expression change.

We were able to validate cyclin D1 upregulation in WDR73-suppressed podocytes by RT-qPCR (Fig. 4Ai), and by western blot (Supplementary Fig. S7). Interestingly, we also observed significantly increased expression of *CCND1* in INTS11-depleted podocytes (Fig. 4Ai). In addition, we were able to validate by RT-qPCR an upregulation of genes encoding the other G1 phase cyclins, cyclin D2 (*CCND2*) and cyclin D3 (*CCND3*), following WDR73 knockdown, and decreased expression of the cyclin-dependent kinase inhibitor *CDKN1A* (Fig. 4Aii–iv). *CDKN1A* encodes the protein p21^Cip1^, which functions to oppose cyclin D1 activity and negatively regulate G1 progression^28^. We found the same effect in INTS11-suppressed cells for *CCND3*, *CCND2* and *CDKN1A* as was observed for WDR73 KD (Fig. 4). Most interestingly, in performing Ingenuity pathway analysis of the list of genes up- and downregulated in WDR73-depleted cells compared to control, we noticed, among the canonical pathways identified, an enrichment of genes encoding proteins involved in “G1/S checkpoint regulation” (Supplementary Fig. S6B). We were intrigued to note that, for the large part, genes whose downregulation is expected to promote G1-S transition (such as *CCND1, CCND2* and *CCND3* but also *BMI1*, *E2F5*, *HDAC4, HDAC9* and *MYC*) were upregulated in WDR73-suppressed cells. Inversely, a number of genes whose upregulation is expected to promote G1-S transition (including *CDKN1A* but also *CDKN2B*, *CDKN2C* and *CDKN2D*) were downregulated following WDR73 knockdown (Fig. 4B).

In order to further characterise the role of WDR73 on cell cycle progression, we suppressed WDR73 using siRNA in immortalised podocytes, and quantified the proportion of cells in either G1, S, G2/M using propidium iodide staining and flow cytometry. We observed a trend for the proportion of WDR73-suppressed cells in S phase to increase, with no observable change on the proportion of cells in G2/M phase (Supplementary Fig. S8Ai–iii,Bi,ii). This finding was supported by our result that WDR73-depletion had no effect on the proportion of phospho-histone H3 (pH3)-positive cells, this being a marker which identifies actively dividing cells (Supplementary Fig. S8C).

Altogether, these results imply that reduced expression of WDR73 is associated with deregulated expression of genes encoding cell-cycle regulatory proteins, notably those which encode proteins that promote G1/S phase transition.

## Discussion

In this study, we have demonstrated association of WDR73 with the INTS9 and INTS11 components of the Integrator complex. We have shown that in cells in which WDR73 expression is either absent or reduced, cellular processes known to be regulated by the Integrator complex, including UsnRNA 3′ processing and EGF responsiveness, are perturbed. We have recently shown that disruption of tRNA post-transcriptional modification pathways underlie disease development in GAMOS patients with mutations in genes encoding KEOPS complex components, and so that we report here UsnRNA misprocessing in patient-derived NPCs supports the emerging consensus that deregulation of RNA metabolism is common feature of GAMOS pathogenesis^29,30^.

Previous work demonstrating localisation of WDR73 to microtubules, and immunoprecipitation of WDR73 with tubulin proteins, prompted hypotheses that the function of WDR73 was to regulate microtubule dynamics^3,4^. In our proteomic analyses, we were unable to confirm TUBA1B and TUBB4B as WDR73-interacting proteins. However, our experiments were performed under different conditions in a different cell type, which could account for this discrepancy. Due to our exciting identification of INTS9 and INTS11 as WDR73-associating proteins, we focussed our investigation on assessing whether WDR73 functions in the same cellular pathway as Integrator. However, our findings by no means negate previous studies implicating WDR73 in the maintenance of cytoskeletal integrity. Indeed, our results suggest that WDR73 may participate in regulation of transcriptional initiation downstream of growth factor stimulation. Like the majority of cellular proteins, genes encoding cytoskeletal components and their associated regulators are subject to strict transcriptional control, deregulation of which may also cause generalised cytotoxic stress with secondary effects on the cytoskeleton^31,32^.

We have here demonstrated an upregulation of *CCND1, CCND2* and *CCND3*, encoding cyclin D1, D2 and D3 respectively, and a downregulation of *CDKN1A,* which encodes p21^Cip1^, following WDR73 knockdown. It therefore appears that WDR73 suppression is linked to deregulation of genes encoding regulators of the G1/S checkpoint transition, a hypothesis supported by our Ingenuity pathway analysis on RNA-Seq data from WDR73-suppressed cells. The most well described function of cyclin D1 is as a cyclin-dependent kinase-4 (CDK4) and CDK6 activator^27,33^. A key function of p21^Cip1^ is to repress the activity of several cyclin-CDK complexes, including cyclin D1-CDK4/6, and so *CDKN1A* downregulation also promotes cell cycle progression through G1 and into S phase^34^. However, we observed no significant changes in the proportion of cells in each cell-cycle phases, no differences in pH3 staining and no changes in the expression of cyclins which regulate progression through later stages of the cell cycle by RNA-Seq, in WDR73 depleted cells compared to control. We can thus conclude that although WDR73 suppression may lead to deregulated G1/S phase progression, we would not expect WDR73 loss to promote cell proliferation. Our results are therefore consistent with previous findings that *WDR73* mutation causes a reduction in cell cycle progression with fewer actively dividing cells^3,4^. Of note, Ben-Omran et al.(2015) report that actively dividing NPCs in the brains of *wdr73* morphant zebrafish persist abnormally in a proliferative state and exhibit increased levels of apoptosis, implying some problem with cell cycle progression.

Based on our findings implicating WDR73 in cell cycle control, we hypothesise that in highly specialised cells such as neurons and podocytes, WDR73 acts to maintain cells in a differentiated state and inhibit either cell cycle re-entry or progression. It appears that WDR73 is largely unessential for development, as the brains and kidneys of GAMOS patients with *WDR73* mutations develop normally^3^. Rather, *WDR73*-linked GAMOS manifests as a degenerative disorder, and so the hypothesis that WDR73 has a role in maintaining cellular quiescence is consistent with this presentation of disease. Reduced levels of WDR73 protein such as occurs in GAMOS patients with loss of function *WDR73* mutations, might therefore cause aberrant cell cycle re-entry. Indeed, although both podocytes and neurons are post-mitotic, they retain the ability to re-enter the cell cycle^35^. This phenomenon occurs under physiological conditions, and is thought to permit podocytes to cope with glomerular injury^36,37^. However, it is critical that cell cycle progression is halted before the G2/M checkpoint, as podocytes and neurons are unable to form a spindle, and will die by mitotic catastrophe if they attempt to do so^38,39^. WDR73 suppression appears not to affect the proportion of cells persisting in G2/M, however, it may be that failure to arrest at an earlier checkpoint would also result in apoptosis. It has been demonstrated in a cell culture model that cell cycle re-entry sensitises podocytes to death by secondary injuries^40^. Perhaps similarly, WDR73 loss makes podocytes more susceptible to chemical and physical insults, subsequently leading to apoptosis and detachment from the glomerular basement membrane. Moving forward, it would be interesting to further explore whether WDR73 loss is associated with a reduced capacity of differentiated cells to maintain their identity, or encourages bypass of the G1/S checkpoint that under normal physiological conditions would prevent attempts of the podocyte or neuron to undergo mitosis.

We report here an upregulation in the mRNA encoding cyclin D1 following WDR73 depletion. A review of the literature reveals different glomerular diseases are associated with variable changes in cyclin D1 levels, largely depending on whether the disease presents with a proliferative or non-proliferative phenotype. For example, cyclin D1 expression is decreased in cases of classic focal segmental glomerulosclerosis (FSGS), and increased in cases of HIV-associated nephropathy, chronic glomerulonephritis (CGN) and collapsing FSGS. Expression levels of p21^Cip1^ are also differentially affected depending on the nature of the renal disease in question, yet are reported to be decreased in the glomeruli of children with collapsing glomerulopathy^41^. Our data on mRNA levels of cyclin D1 and p21^Cip1^ are consistent with our report that the renal lesions in patients affected by *WDR73*-linked GAMOS are of the collapsing type^3^. Recently, collapsing FSGS has also been reported in two GAMOS patients with homozygous missense mutations in *WDR73*^11^.

Further in regard to *CCND1* upregulation in WDR73-depleted cells, it is perhaps also worth considering how non-canonical roles of cyclin D1, independent of CDKs, may impact GAMOS pathogenesis^42^. For example, cyclin D1 is able to interact with the C-terminal domain of RNAPII, and its overexpression has been linked to global transcriptional down-modulation as a result of a negative effect on RNAPII pause-release^43^. As regulation of RNAPII promoter proximal pausing is also one of the principal roles attributed to the Integrator complex^22,23^, we postulate that perhaps a combined effect of Integrator complex dysfunction and cyclin D1 overexpression is responsible for the defect in EGF-early gene induction observed here in WDR73-depleted podocytes. Moving forward, it would be interesting to assess whether levels of paused RNAPII are altered at promoter proximal sites in WDR73-depleted cells using chromatin immunoprecipitation sequencing.

As previously mentioned, mutations in genes encoding Integrator complex components INTS1 and INTS8 are associated with the development of a microcephalic neurological disorder reminiscent of that affecting GAMOS patients with *WDR73* mutations^15^. Patients with mutations in *INTS1* and *INTS8* are affected by profound intellectual disability, borderline microcephaly, cerebellar hypoplasia and reduced volume of the pons and brainstem. Most interestingly, fibroblasts from patients harbouring *INTS8* mutations have also been shown to contain elevated levels of *CCND1* transcripts compared to control. No overt kidney phenotype has yet been linked to Integrator complex dysfunction, although one patient with *INTS1* mutation was reported to have renal dysplasia. In addition, it is known that mutations in genes encoding a variety of proteins which act downstream of Integrator in various aspects of UsnRNA and mRNA metabolism are responsible for a range of phenotypically related neurological disorders. For example, mutations in *RNU4atac*, encoding a UsnRNA component of the U12-dependent spliceosome, may result either in microcephalic osteodysplastic primordial dwarfism type I (MOPDI) or Taybi-Linder syndrome, rare conditions characterised by intrauterine and post-natal growth restrictions, microcephaly and cerebellar hypoplasia^44,45^. Taking into consideration the phenotypical similarity of these conditions with our identification of WDR73 as an Integrator-associating protein, we can suppose that Integrator, WDR73 and the aforementioned small RNAs and RNA-metabolising proteins act in overlapping and converging cellular pathways, perturbations in which underlie a range of related microcephalic diseases.

We have demonstrated reduced induction of a subset of EGF-early genes in WDR73-depleted cells following EGF stimulation. Aberrant activation of the EGF signalling pathway in podocytes is thought to be damaging, with *EGFR* deletion in this cell type attenuating diabetic nephropathy^46^. However, it may be that WDR73 also allows podocytes to mediate a rapid transcriptional response to more physiologically relevant stressors, such as changes in fluid flow shear stress or tension^47^. In addition, based on our finding by RNA-Seq that WDR73 suppression is also associated with upregulation of certain genes, we can suppose that if WDR73 participates in promoting RNAPII pause-release, this is not its only function, and WDR73 loss-of-function may be more associated with more widespread and general transcriptional deregulation. In addition, new functions are continually being attributed to Integrator, and it may be that WDR73 participates in regulating a new, as yet uncharacterised, activity of the Integrator complex.

In this study, we have identified and described an interaction of WDR73 with the Integrator complex, and found *WDR73* suppression or mutation to result in alterations in pathways known to be mediated by Integrator. Perturbations in these pathways likely contributes to the development of disease in patients affected by *WDR73* mutations. Our results do not allow elucidate precisely how Integrator activity may be modulated by WDR73, or vice versa, and suggest that perhaps different mechanisms of pathogenicity are at play in different cell types. Further investigation is thus required to fully understand how defects in the spliceosomal and transcriptional machinery differentially affect various cell types, and why highly specialised post-mitotic cells such as neurons and podocytes appear to be so disproportionately affected by deregulation of the spliceosome and RNA processing machinery.

## Materials and methods

### Antibodies

Primary antibodies used in this study include: INTS11 (BETHYL, cat. # A301-274A, western blot (WB): 1/1000), INTS9 (Prestige antibodies, SIGMA, cat. # HPA051615, WB: 1/1000), GFP (ROCHE clones 7.1 and 13.1, cat # 11814460001, WB: 1/2000), WDR73 (Prestige antibodies, SIGMA, cat # HPA039357, WB: 1/1000, immunofluorescence: 1/200), INTS4 (BETHYL, cat. # A301-296A, WB: 1/1000), HDAC1 (CALBIOCHEM, cat. # PC544, WB: 1/1000), actin (SIGMA, cat. # A2228, WB: 1/2000), tubulin (SIGMA, cat. # T8328, WB: 1/2000), EGFR (ABCAM, cat. #ab52894, WB: 1/1000), phospho-histone H3 (clone D2C8, CELL SIGNALING TECHNOLOGIES, cat. # 3377S, flow cytometry: 1/1600), GAPDH (MILLIPORE, cat#MAB374, WB: 1/2000) and cyclin D1 (CELL SIGNALING TECHNOLOGIES, cat#2978T, WB:1/1000). Secondary antibodies used in this study include horseradish peroxidase conjugated mouse and rabbit IgG (GE HEALTHCARE, cat #A931 and A934, WB: 1/10,000) and Alexa-488 conjugated anti-rabbit IgG (LIFE TECHNOLOGIES, IF: 1/400).

### Plasmids

cDNA encoding human WDR73 was purchased from ORIGENE (cat. # RG209040), amplified by PCR, and then subcloned into a LentiORF pLEX-MCS expression vector (OPEN BIOSYSTEMS) previously modified to contain a green fluorescent protein (GFP) tag using the restriction sites NheI and NotI to create the construct pLEX-GFP. Plasmids encoding the WDR73 amino- and carboxy-terminal regions comprising amino acids 1-255 and 256-378 respectively were amplified by PCR from the pLEX-GFP-WDR73 construct, and subcloned back into pLEX-GFP between the NheI and NotI restriction sites. cDNA clones for INTS9 and INTS11 were purchased from SOURCE BIOSCIENCE (sequence IDs. MGC:39162 and MGC:14999 respectively) and again subcloned between the NheI and NotI sites of pLEX-GFP. The regions encoding INTS11 fragments 1-207, 1-390, 1-450 and 1-493 were amplified from pLEX-GFP INTS11 and subcloned back into pLEX-GFP, again using the restriction sites NheI and NotI. Mutations encoding the protein variants WDR73 p.Arg256Profs*18 and INTS11 p.L509A were introduced into pLEX-GFP WDR73 and pLEX-GFP INTS11 respectively using the Q5 site-directed mutagenesis kit (NEW ENGLAND BIOLABS) according to manufacturer’s instructions. The sequences of all constructs were verified by Sanger sequencing. All primers used for subcloning and site-directed mutagenesis reactions are listed in Supplementary Table S8.

### Cell culture and establishment of lentiviral cell lines

The conditionally immortalised human podocyte cell line (AB8/13) used in this study was kindly provided by M.A. Saleem (University of Bristol, Southmead Hospital)^48^. Lentiviral particles containing the pLEX-GFP WDR73 construct were produced by the lentivector production facility Structure Fédérative de Recherche BioSciences Gerland-Lyon Sud (UMS3444/US8). To establish a line of immortalised podocytes stably expressing either GFP or GFP-WDR73, cells were transduced at a multiplicity of infection (MOI) of 2 and then subjected to puromycin selection (2 µg/ml). All podocyte cell lines were cultured in RPMI 1640 media supplemented with 10% foetal calf serum (FCS), 1% penicillin/streptomycin, 2 mM glutamine and 1% insulin/transferrin/selenium at 33 °C, 7% CO_2_.

HEK293T cells (ATCC CRL-3216) were routinely cultured in DMEM 10% FCS supplemented with 1% penicillin/streptomycin and glutamine at 37 °C, 5% CO_2_. Neural progenitor cells (NPCs) were cultured in neural induction media (50% Neurobasal media, 50% DMEM/F-12 plus GlutaMAX (both GIBCO), supplemented with 2% NeuroCult SM1 without Vitamin A, 1% N2 Supplement-B (both STEMCELL TECHNOLOGIES), 1% penicillin/streptomycin, 10 ng/ml fibroblast growth factor 2, 10 ng/ml epidermal growth factor and 20 ng/ml brain-derived neurotrophic factor. Cell culture plates, flasks and glass coverslips (for IF experiments) for NPCs were coated first with Poly_-L_-Ornithine diluted 1/6 in cell culture grade PBS at 37 °C for at least 6hrs. After this time, the cell culture plates were washed 2× in PBS, then coated in laminin (INVITROGEN, cat. # 23017-015) diluted 1/500 in PBS. After coating with the laminin solution for at least 4hrs, the solution was removed and the cells plated were directly onto the coated surface. NPC cell lines derived from healthy persons and from the GAMOS patient are unrelated to each other. All cell lines were determined mycoplasma-free by PCR testing.

### Transfection of cells with siRNA and plasmids

Transfection of DNA plasmids into HEK293T cells was performed using FuGene HD (PROMEGA) according to manufacturer’s instructions. Briefly, DNA and the reagent were mixed at a ratio of 1:3 in OptiMEM (GIBCO), allowed to incubate for 15 min at room temperature before being added to cells grown in complete media. Cells were collected for biochemical analysis either 24 or 48 h following transfection.

ON-TARGETplus siRNA SMARTpools targeting WDR73 and INTS11 were purchased from DHARMACON (cat. #. L-01524-02 and L-013789-01 respectively). In each siRNA experiment, cells transfected with a non-targeting oligonucleotide (sequence 5′-GUUAUGUCGAACAUUGAUCAU-3′) were included as a negative control. Cells were transfected with siRNA at a final concentration of 12 nM using Lipofectamine RNAi max (LIFE TECHNOLOGIES) according to manufacturer’s instructions. Briefly, the required quantity of siRNA and reagent were separately diluted in OptiMEM and incubated for 5 min before mixing and a further incubation for 20 min. The mixture was added to the cells and media changed 4 h later. Cells were collected for analysis either 72 or 96 h following transfection.

### EGF-stimulation experiments

24 h preceding stimulation, media was removed and cells washed 1× in PBS before media was replaced with complete media supplemented with 0.1% FCS. Cells were then stimulated with EGF for 30 min at a final concentration of 100 ng/ml before isolation for subsequent analysis.

### Cell lysis and western blotting

Cells were routinely lysed in buffer composed of 50 mM Tris–HCl (pH7.4), 150 mM NaCl, 1 mM EDTA, 1% Triton X-100 and 1 tablet protease inhibitor (ROCHE) per 10 ml. Following lysis, cells were incubated on ice for 10 min before centrifugation at 12,500 rpm for 10 min at 4 °C. Protein concentration was determined using BCA assay, and then equal amounts of protein per condition were diluted in 4× sample buffer (BIO-RAD) supplemented with β-mercaptoethanol. Samples were then loaded onto pre-cast PROTEAN any kDa polyacrylamide gels (BIO-RAD). Following migration, proteins were transferred onto a nitrocellulose membrane, and subjected to standard immunoblotting (briefly, blocking in Tris-buffered saline 0.1% Tween-20 (TTBS), 5% milk for 1 h, followed by overnight incubation in primary antibody diluted in TTBS 5% bovine serum albumin (BSA), then 3× TTBS 0.1%Tween washes, incubation with horseradish peroxidase-coupled secondary antibodies (GE HEALTHCARE) diluted 1/10,000 in TTBS 5% milk for 1 h at room temperature followed by a final three washes). Proteins were revealed using ECL and Fusion chemiluminescence imaging system. Protein band intensity was quantified using BioID software.

### Immunoprecipitation

For immunoprecipitation experiments (IP), cells were lysed in the required volume of lysis buffer (as used for standard cell lysis), then incubated on ice for 10 min before centrifugation at 12,500 rpm for 4 °C a further 10 min. Following this step, 10% of the lysate volume was removed and diluted in an equal volume of 4× sample buffer supplemented with β-mercaptoethanol. For IP of tagged proteins, the remaining supernatants were then added to a separate tubes containing 30–50 µl of anti-GFP beads (MILTENYI BIOTEC) and incubated for 2hrs at 4 °C with rotation. After 2hrs, beads were loaded onto µMACs separation columns (MILTENYI BIOTEC), washed 3× in lysis buffer and 1× in 20 mM Tris–HCl pH7.4 before elution into 70 µl hot 2× sample buffer supplemented with β-mercaptoethanol. For IP of endogenous proteins, the lysates were incubated overnight either with 1–2 µg of primary antibody as required, or an equivalent quantity of isotype control immunoglobulin. The following day, between 30 and 50 µl of either Protein-A or Protein-G beads, as required (MILTENYI BIOTEC), were added and samples rotated at 4 °C for a further 2 h. Washing and elution were carried out as for IP of tagged proteins.

### RNA extraction, reverse transcription and quantitative-PCR

RNA was extracted from cells using the RNA easy mini kit (QIAGEN) according to manufacturer’s instructions. 0.6 µg RNA per condition was subjected to DNase digestion and then reverse-transcribed. 100 ng cDNA per well was used in subsequent quantitative PCR (qPCR) experiments performed using SYBR Green master mix (LIFE TECHNOLOGIES). All primers for qPCR experiments are shown in Supplementary Table S9. See supplementary methods for note on primer design for U12 and SNORD3A.

### Statistical analyses

GRAPHPAD PRISM 5.0 software (La Jolla, California) was used for the graphical representation and statistical analysis. Data are presented as mean ± S.E.M of at least *n* = 3 independent experiments. To test if the difference between two experimental conditions in which data had not been normalised was significant, the non-parametric Mann–Whitney U test was applied. If comparisons were being made between more than two conditions, a one-way ANOVA followed by either the Bonferroni multiple comparisons or Dunnett post-hoc test was performed. In cases where data had been normalised (in qPCR experiments, for example), unless otherwise stated, a one sample t-test was performed. Y axes of graphs are logarithmic (log2) in the following figures: Fig. 2, panels A to C, Fig. 4, panels Aii to Aiv.

### Ethics declaration regarding use of human cell lines

Written informed consent was obtained from all participants. All methods were performed in accordance with relevant guidelines and regulations. Methods of sample collection, storage and experimental protocols for induced human pluripotent stem cells (iPSCs) and their derivatives were declared to the French research ministry (identification DC 2015-2595) and have obtained approval from the ethical Committee "CPP Ile-de-France II" on September 5th, 2016.

## Supplementary Information

Supplementary Information.

Supplementary Table S1.

Supplementary Table S2.

Supplementary Table S3.

Supplementary Table S4.

Supplementary Table S5.

Supplementary Table S6.

Supplementary Table S7.

## Data Availability

Most data generated and/or analyzed during this study are included in this published article (and its Supplementary Information files). Datasets not included are available from the corresponding author on reasonable request.
