# Supplementary material for: Disruption of pathways regulated by Integrator complex in Galloway–Mowat syndrome due to WDR73 mutations
Source: Sci Rep. 2021 Mar 8;11:5388. doi: 10.1038/s41598-021-84472-7 (PMC7940485; doi:10.1038/s41598-021-84472-7)
Supplement: Supplementary file 1 — Supplementary Information. [file 41598_2021_84472_MOESM1_ESM.pdf]

SUPPLEMENTARY INFORMATION

Disruption of pathways regulated by Integrator complex in Galloway-Mowat syndrome due to *WDR73* mutations

Tilley, F.C.[1], Arrondel, C[1], Chhuon, C.[2], Boisson, M.[1], Cagnard, N.[3], Parisot, M.[4], Menara G.[1], Lefort, N.[5], Guerrera, I.C.[2], Bole-Feysot, C.[4], Benmerah, A.[1], Antignac, C[1,5], Mollet, G.[1]*

Content:

Supplementary Figures:

Supplementary Figure S1: Identification of WDR73-interacting proteins

Supplementary Figure S2: Subcellular localisation of WDR73

Supplementary Figure S3: Characterisation of the WDR73-Integrator complex interaction

Supplementary Figure S4: *WDR73* and *INTS11* knock-down efficiencies in podocytes related to Fig.2A.

Supplementary Figure S5: Effects of *WDR73* suppression on EGFR protein levels and transcriptional regulation in immortalized podocytes

Supplementary Figure S6: Ingenuity pathway analysis of the genes significantly differentially expressed in *WDR73*-suppressed cells compared to control siRNA-treated cells with no EGF stimulation

Supplementary Figure S7: Effect of *WDR73* suppression on cyclin D1 protein level in podocytes

Supplementary Figure S8: Effect of *WDR73* suppression in podocytes on the number of cells in the G1, S and G2 cell cycle phases

Supplementary Figure S9: Uncropped blots

Supplementary Tables:

Supplementary Tables S1-S7 are provided as separated excel files.

Supplementary Table S8: Primers used for subcloning and site-directed mutagenesis reactions

Supplementary Table S9: Primers used in quantitative PCR experiments

Supplementary Methods:

Generation and quality control of neural progenitor cells

Nucleo-cytoplasmic fractionation

Proteomic analysis

Design of qPCR primers for U12 and SNORD3A

RNA-Sequencing and subsequent analysis

Flow cytometry

Immunofluorescence

Supplementary tables:

Supplementary Table S1: Proteins identified by mass spectrometry of GFP-immunoprecipitates isolated from a human podocyte cell line stably expressing GFP-WDR73.

Supplementary Table S2: Genes identified as being differentially expressed to a significantly different extent by RNA-Seq in WDR73 KD cells + EGF compared to Ctrl siRNA-treated cells + EGF (fold change 1.2).

Supplementary Table S3: Full list of genes differentially expressed in WDR73 KD cells + EGF compared to Ctrl siRNA-treated cells + EGF.

Supplementary Table S4: Genes identified as being differentially expressed to a significantly different extent in Ctrl siRNA-treated cells + EGF compared to Ctrl siRNA-treated cells - EGF (fold change 2).

Supplementary Table S5: Full list of genes differentially expressed in Ctrl siRNA-treated cells + EGF compared to Ctrl siRNA-treated cells - EGF.

Supplementary Table S6: Genes identified as being differentially expressed to a significantly different extent in WDR73 KD cells - EGF compared to Ctrl siRNA-treated cells - EGF (fold change 1.2).

Supplementary Table S7: Full list of genes differentially expressed in WDR73 KD cells - EGF compared to Ctrl siRNA-treated cells – EGF.

Supplementary Table S8: Primers used for subcloning and site-directed mutagenesis reactions.

Supplementary Table S9: Primers used in quantitative PCR experiments.

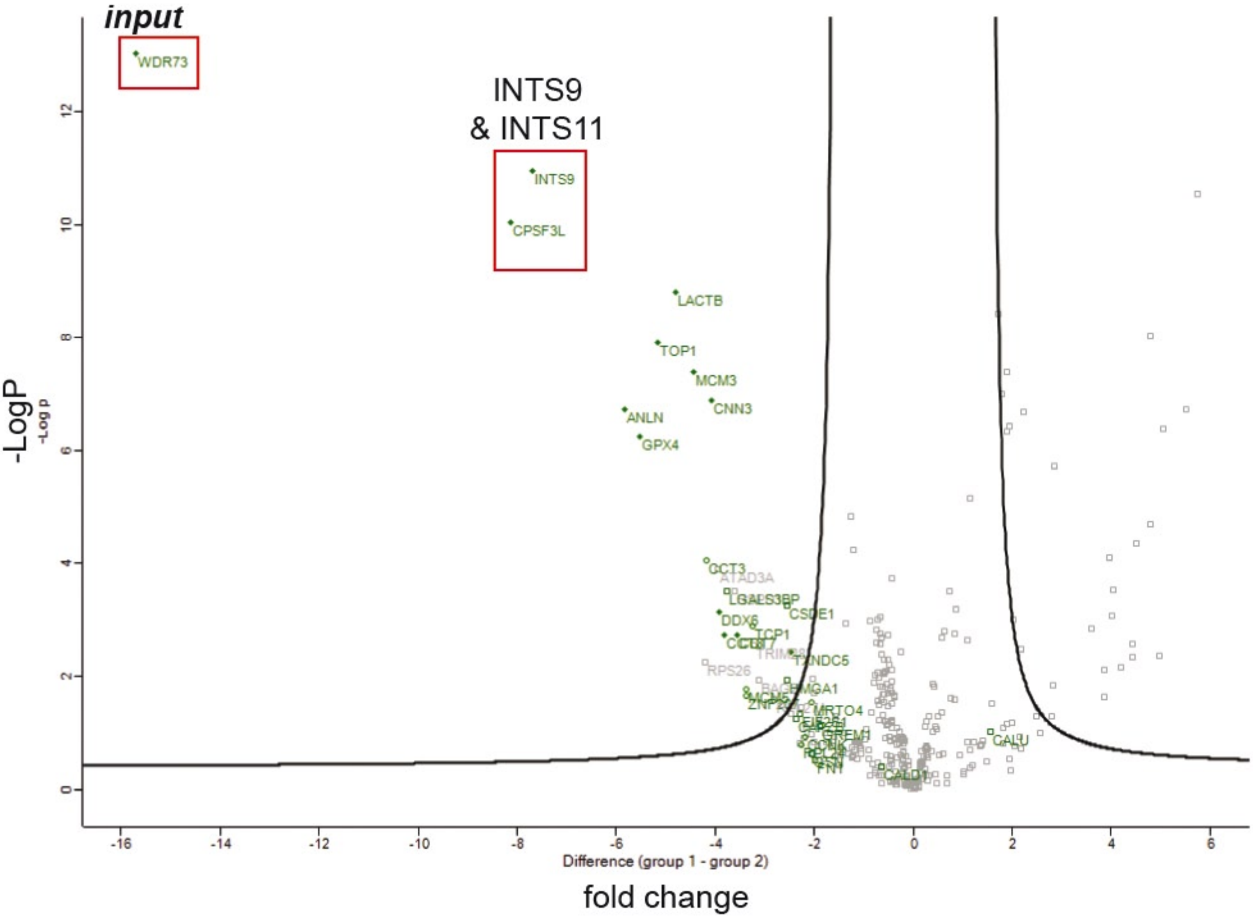

Supplementary Figure S1: Identification of WDR73-interacting proteins.
Volcano plot representation of mass spectrometry analysis of GFP-immunoprecipitates isolated from a human podocyte cell line stably expressing either GFP or GFP-WDR73 wild-type. Proteins which were significantly enriched in the IP from GFP-WDR73 expressing podocytes compared to GFP-only are found in the region left of the central 'volcano'. Red boxes highlight INTS9, INTS11 (CPSF3L) and WDR73. Please note that in this context, there is no significance to the colour in which protein names are shown. Data is representative of six technical repeats.

Supplementary Figure S2

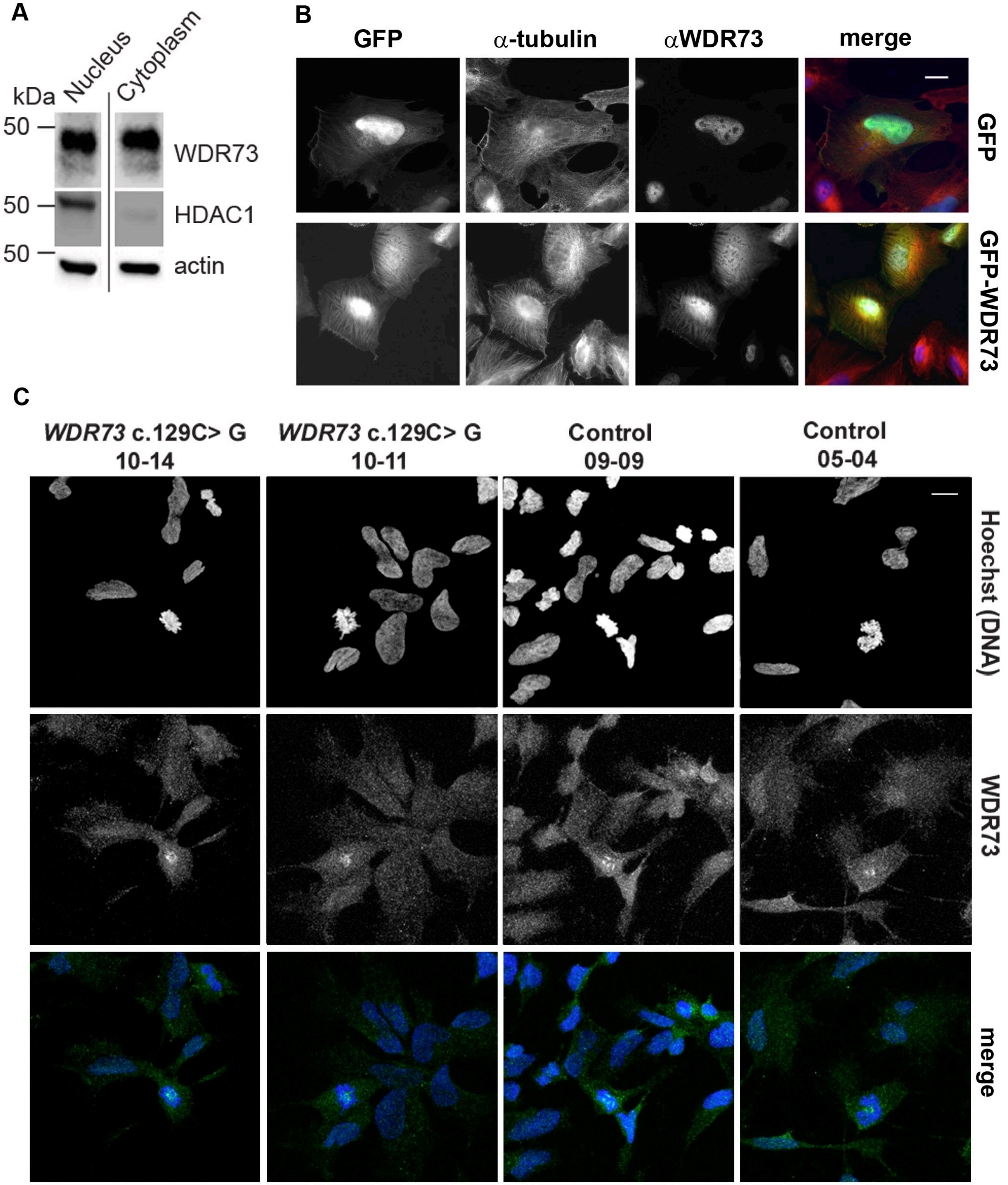

Supplementary Figure S2: Subcellular localisation of WDR73. (A) HeLa cells were subjected to nucleo-cytoplasmic fractionation. The subcellular distribution of WDR73 was assessed using western blotting (WB) with the indicated antibodies. Blot shown is from an image of a single membrane cropped for clarity, and is representative of two independent experiments. **(B)** Immortalized human podocyte cell lines stably expressing GFP or GFP-WDR73 wild-type (cells used in experiments presented in Fig. 1B and 1D). Immunofluorescence shows the subcellular localisation of GFP-WDR73 (GFP column), tubulin (αtubulin), endogenous WDR73 (αWDR73) and the merge. **(C)** IF of endogenous WDR73 in neural progenitor cells (NPCs) derived from two healthy controls (05-04 and 09-09) and two clones derived from a single patient with a c.129C>G mutation in *WDR73* (10-11 and 10-14). This experiment shows that signal from the WDR73 antibody (same as the one used in B) is observed in both control and patient-derived NPCs. As the c.129C>G mutation introduces a premature stop codon encoding a protein of only 43 amino acids, and the antibody used in this staining is raised against a peptide in the C-terminus of WDR73, we have reason to believe this WDR73 antibody is reacting with a non-specific antigen in the patient-derived cells. We have also found siRNA and shRNA-mediated depletion of *WDR73* in a human podocyte cell line to have no effect on signal intensity from this WDR73 antibody by IF, despite disappearance of the signal on western blots (data not shown). The peptide against which this WDR73 antibody is raised does not occur in either of the three potentially protein coding *WDR73* transcripts listed in Ensembl [50]. We have found this antibody to be capable of recognising GFP-WDR73 when overexpressed in a podocyte cell line (shown in B), and other groups have reported spindle localisation of WDR73 using a tagged protein [4], altogether suggesting that some of the antibody IF signal may reflect the true subcellular localisation of endogenous WDR73. However, based on our findings, we suggest caution to other researchers embarking on WDR73 imaging experiments. Scale bar = 10μm.

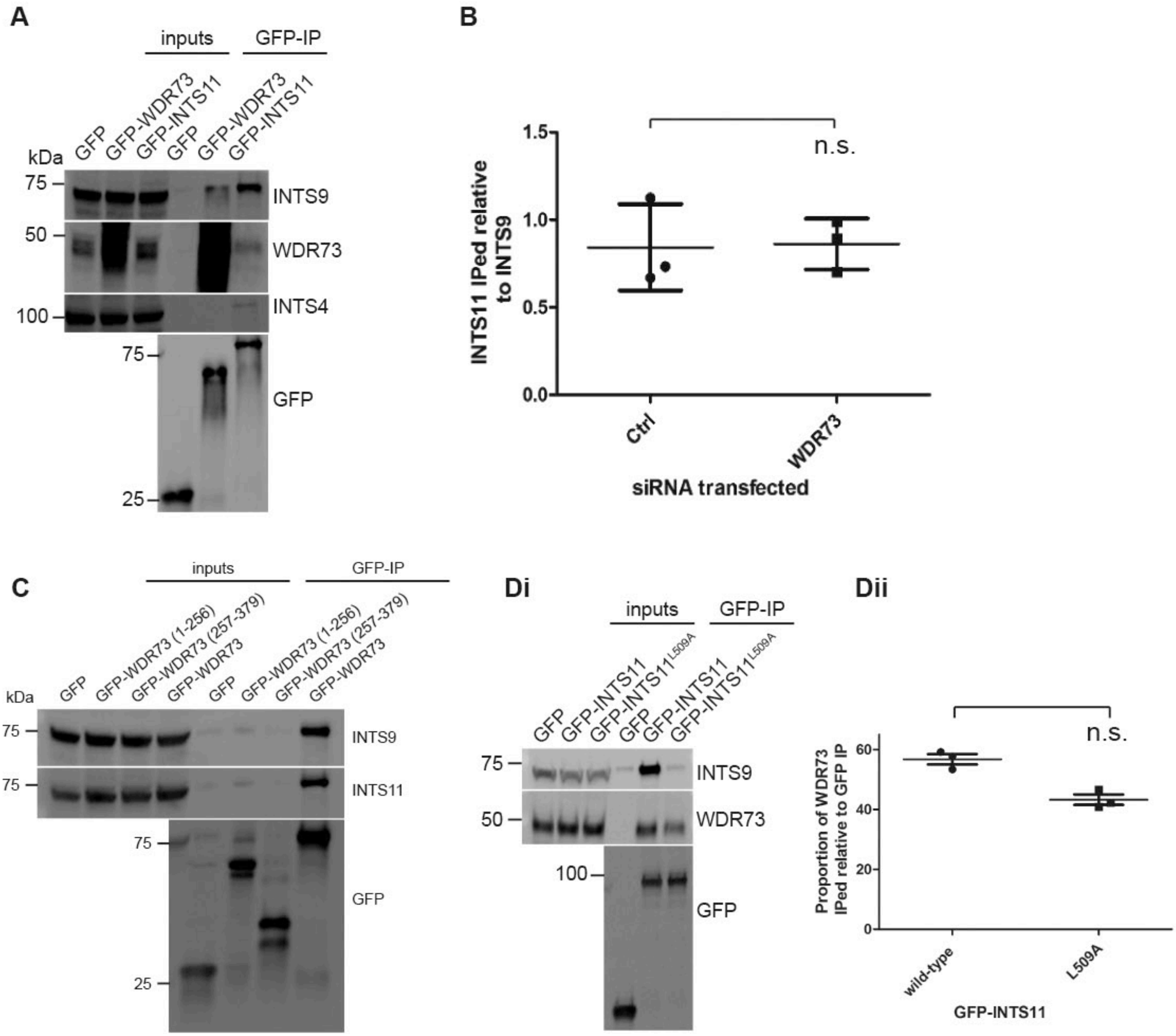

Supplementary Figure S3: Characterisation of the WDR73-Integrator complex interaction.
(A) GFP-IP of the indicated constructs from transiently transfected HEK293T cells followed by WB confirms INTS11-INTS4 association, and a lack of WDR73-INTS4 association. (B) Quantification of data shown in Fig. 1E. Intensity values of INTS9 protein bands in IP lanes were first normalised to values of INTS9 protein band intensity in the corresponding input lane. Intensity values of INTS11 protein bands in the IP lanes were then divided by these normalised INTS9 intensity values. (C) GFP-IP of the indicated constructs from transiently transfected HEK293T cells followed by WB reveals only full length wild-type WDR73 interacts with INTS9 and INTS11. (Di) GFP-IP of the indicated constructs from transiently transfected HEK293T cells shows a trend for diminished association of WDR73 with GFP-INTS11L509A. (Dii) Quantification of data shown in (Di). Intensity values of WDR73 proteins bands in the GFP-IP lanes were divided by intensity values of GFP protein bands in the corresponding lane. All blots and graphs shown in this figure represent data from three independent biological repeats. Error bars are ± S.E.M., n.s. indicates that the difference between conditions is not significant as determined using a Mann-Whitney test.

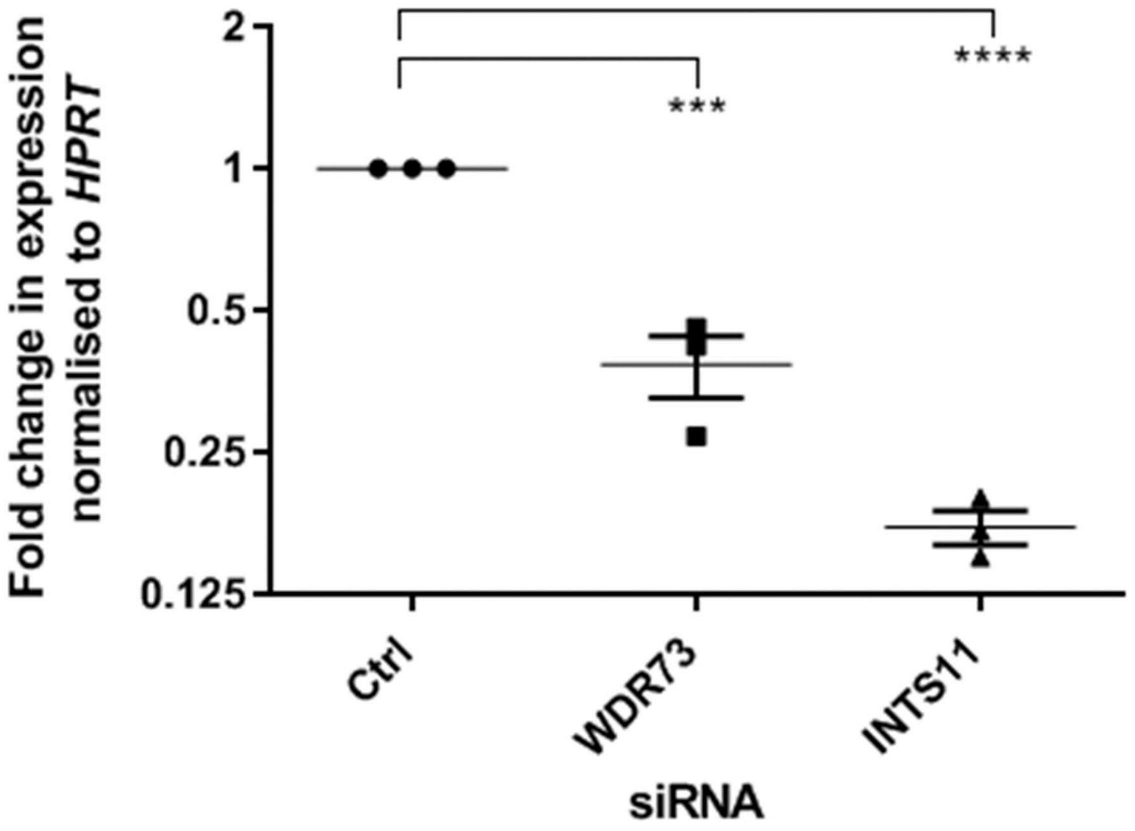

Supplementary Figure S4: *WDR73* and *INTS11* knock-down efficiencies in podocytes related to Fig.2A. RT-qPCR analysis demonstrating normalised *WDR73* and *INTS11* expression following knockdown in the three experiments shown in Fig.2A.

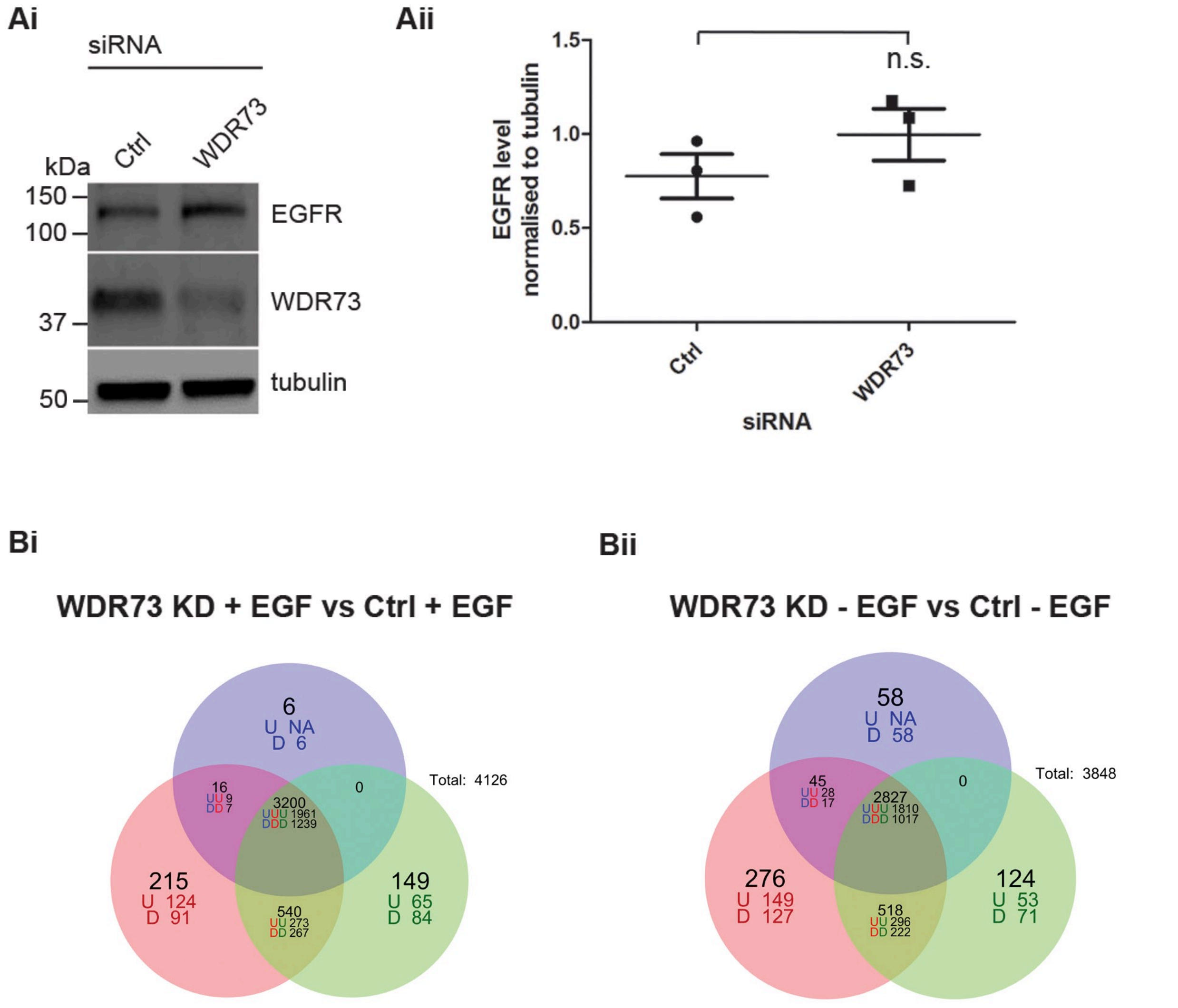

Supplementary Figure S5: Effects of WDR73 suppression on EGFR protein levels and transcriptional regulation in immortalized podocytes.

(Ai) Western blot showing that WDR73 suppression does not impact EGFR protein levels. Blot shown is representative of three independent biological repeats. (Aii) Quantification of data shown in (Ai). Intensity values of EGFR protein bands were divided by intensity values of tubulin protein bands in the corresponding lane. Error bars are ± S.E.M., n.s. indicates that the difference between conditions is not significant as determined by a Mann-Whitney test. (B) Venn diagrams showing the number of genes significantly upregulated (U) and downregulated (D) in WDR73-suppressed (WDR73 KD) cells + EGF compared to control siRNA-treated cells + EGF (Ctrl + EGF) (fold change 1.2, list of genes in Supplementary Table S2) (Bi) and WDR73-suppressed cells compared to control siRNA-treated cells with no EGF stimulation (fold change 1.2, list of genes in Supplementary Table S4) (Bii). Blue, red and green circles and letters indicate the number of genes found to be differentially expressed to a statistically significant extent between the conditions as determined by the DEseq2.0, edgeR.0 and Voom.0.

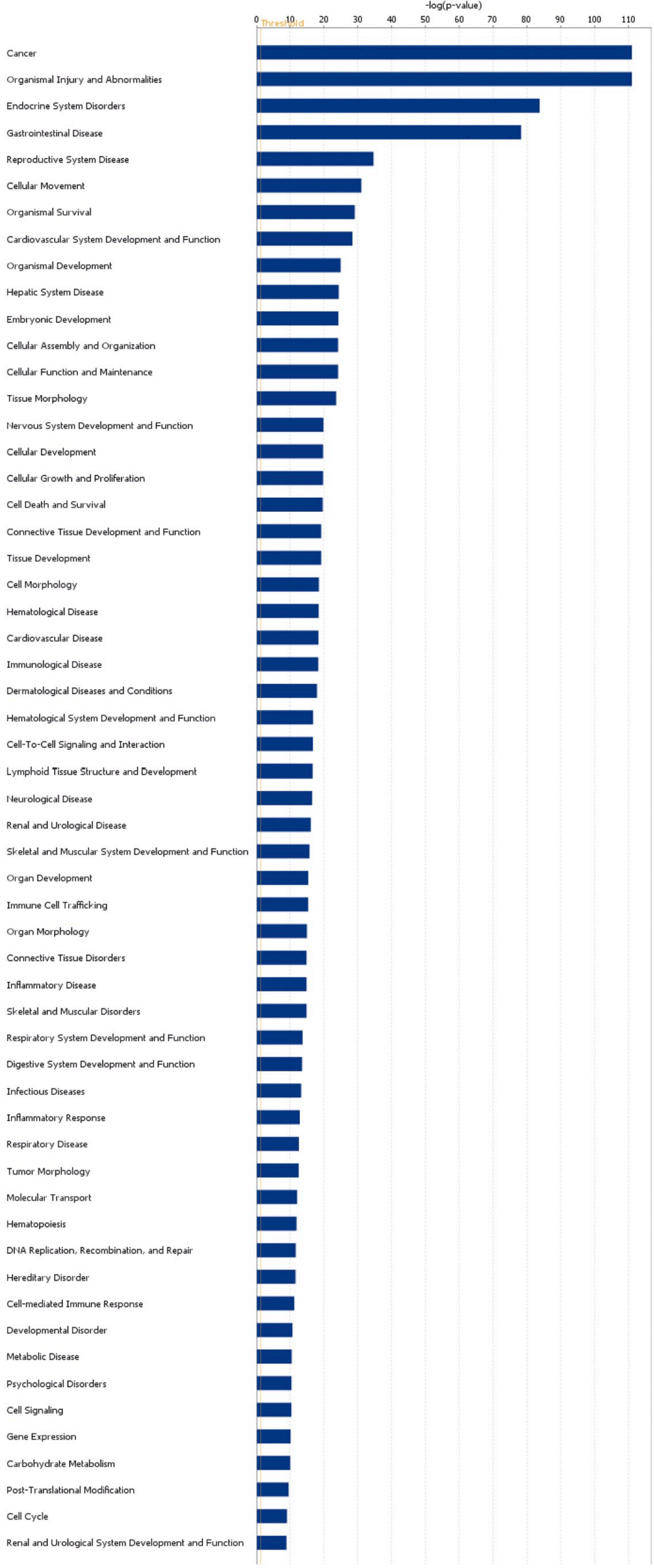

Supplementary Fig S6A

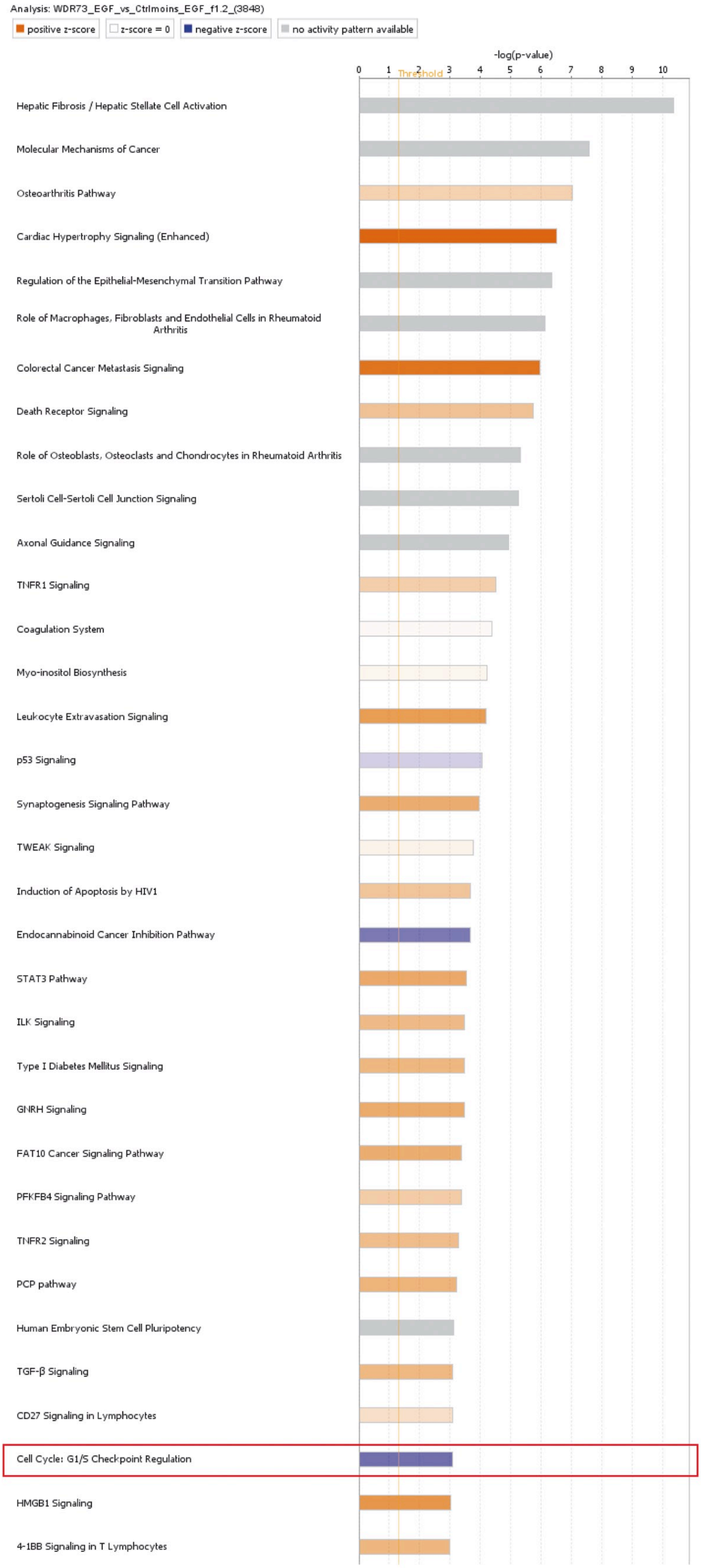

Supplementary Fig S6B

Supplementary Figure S6: Ingenuity pathway analysis of the genes significantly differentially expressed in WDR73-suppressed cells compared to control siRNA-treated cells with no EGF stimulation. Graphical demonstration of Ingenuity Pathway Analysis of associated functions and/or diseases (A) and pathways (B) for the differentially expressed genes listed in Supplementary Table S6. The top significant categories of functions or pathways (y-axis) are shown ordered by significance [-log(p-value)] (x-axis) calculated in Ingenuity pathway analysis by right-tailed Fischers exact t-test. The orange "Threshold" vertical line indicates the cutoff for significance (p-value of 0.05). In (B), an activation Z-score >2 (orange) was defined as the threshold of significant activation, whilst an activation Z-score< -2 (blue) was defined as the threshold of significant inhibition of the pathway. Due to large size of the data file, only the top 34 pathway categories are displayed.

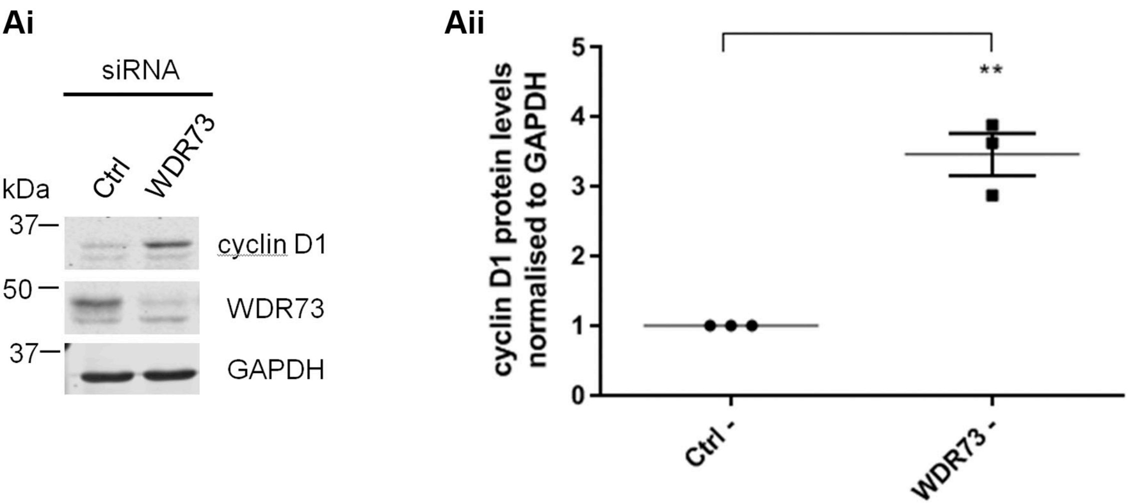

Supplementary Figure S7: Effect of WDR73 suppression on cyclin D1 protein level in podocytes.

(Ai) Western blot showing that WDR73 suppression impacts cyclin D1 protein levels. Blot shown is representative of three independent biological repeats. (Aii) Quantification of data shown in (Ai). Intensity values of cyclin D1 protein bands were divided by intensity values of GAPDH protein bands in the corresponding lane. Error bars are ± S.E.M., n.s. indicates that the difference between conditions is not significant as determined by a unpaired t -test.

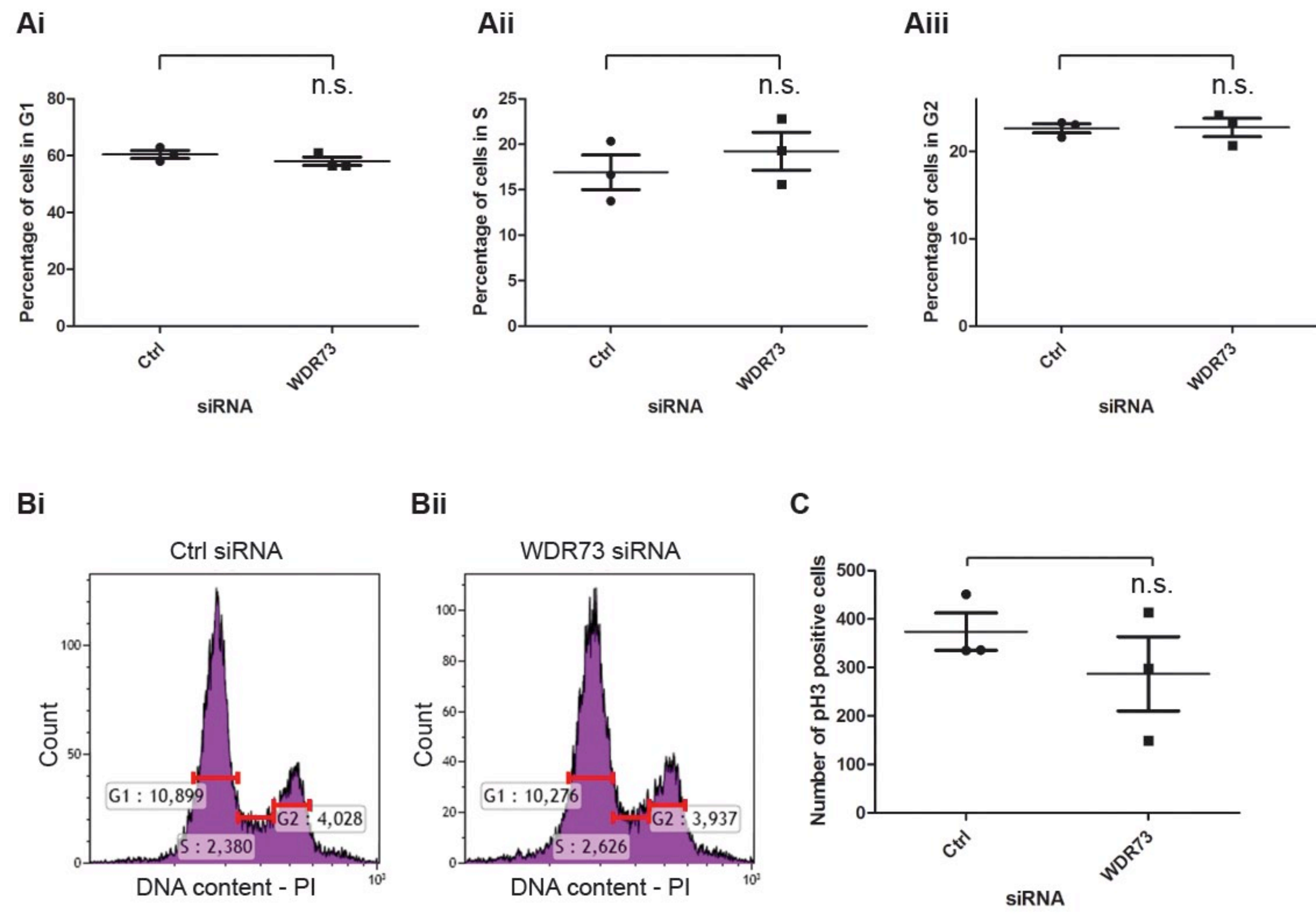

Supplementary Figure S8: Effect of WDR73 suppression in podocytes on the number of cells in the G1, S and G2 cell cycle phases.

(Ai-iii) Propidium iodide (PI) staining of Ctrl siRNA-treated and WDR73-depleted podocytes reveals a trend for the proportion of WDR73 knock-down cells to accumulate in the S phase of the cell cycle. To determine the percentage of cells in each cell cycle stage, gates were drawn over G1 and G2 peaks following forward and side scatter gating, with cells between these two peaks being assigned S-phase status. The number of cells in G1, S and G2 were then divided by the total number of gated cells. (Bi-ii) Representative histograms showing gating strategy used for assigning cell cycle stage to PI-stained Ctrl and WDR73-depleted (WDR73 KD) podocytes. (C) Phospho-histone H3 (pH3) staining of Ctrl siRNA-treated and WDR73-depleted podocytes followed by analysis by flow cytometry reveals no difference in the proportion of actively dividing podocytes between the two conditions. Graphs in this figure show data from three independent biological repeats, error bars are ± S.E.M., n.s. indicates difference between conditions is not significant as determined using a Mann-Whitney test.

Supplementary Figure S9 : Uncropped blots

Uncroped blots corresponding to figure 1B

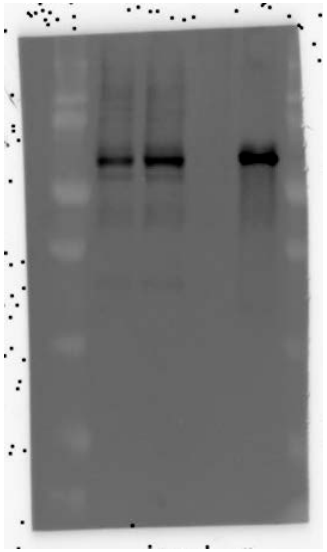

INTS11

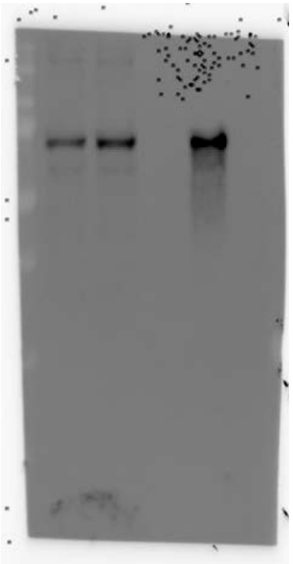

INTS9

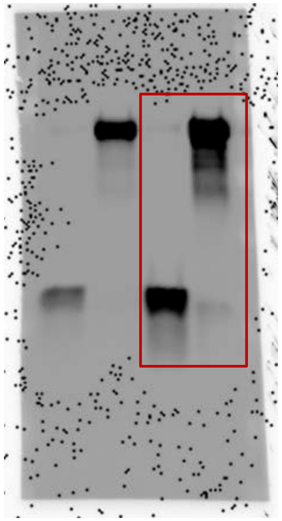

GFP

Uncroped blots corresponding to figure 1Ci

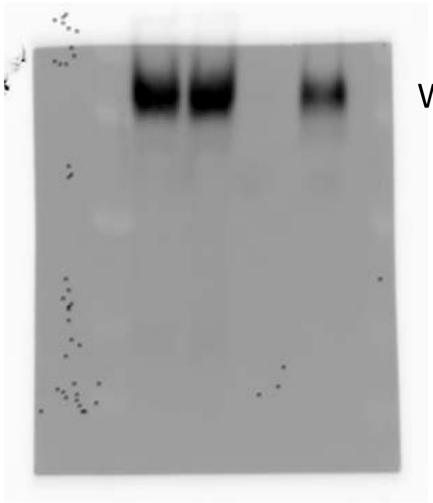

WDR73

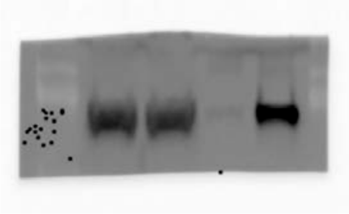

INTS9

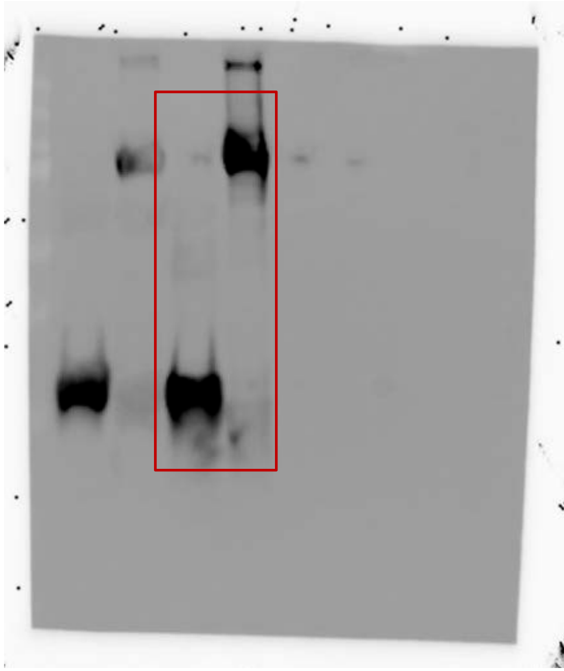

GFP

Uncroped blots corresponding to figure 1Cii

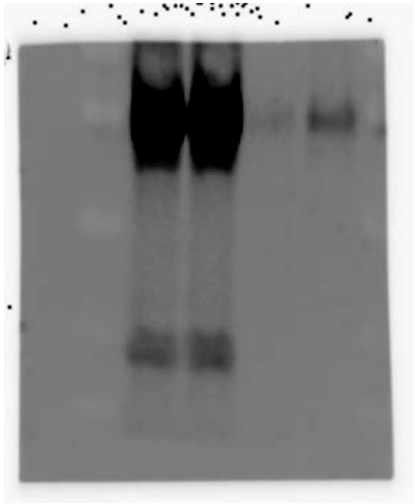

WDR73

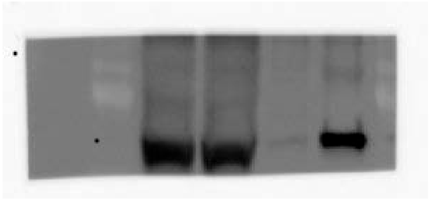

INTS11

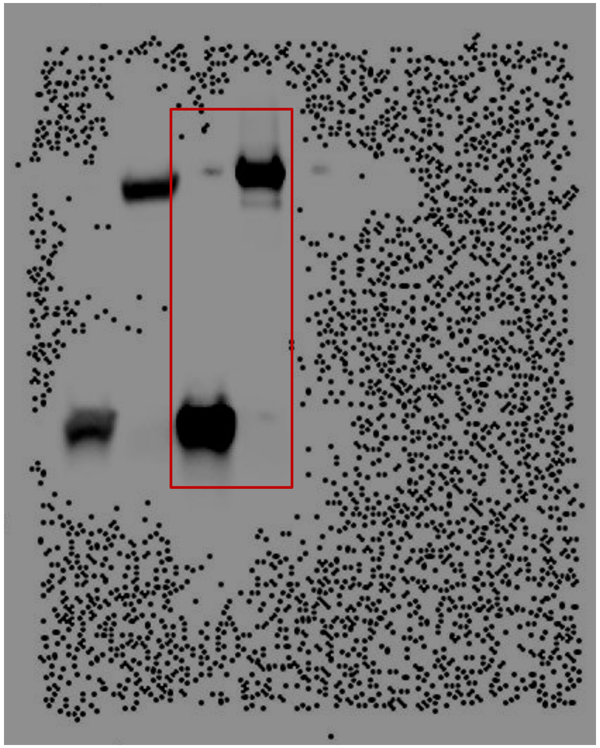

GFP

Uncroped blots corresponding to figure 1D

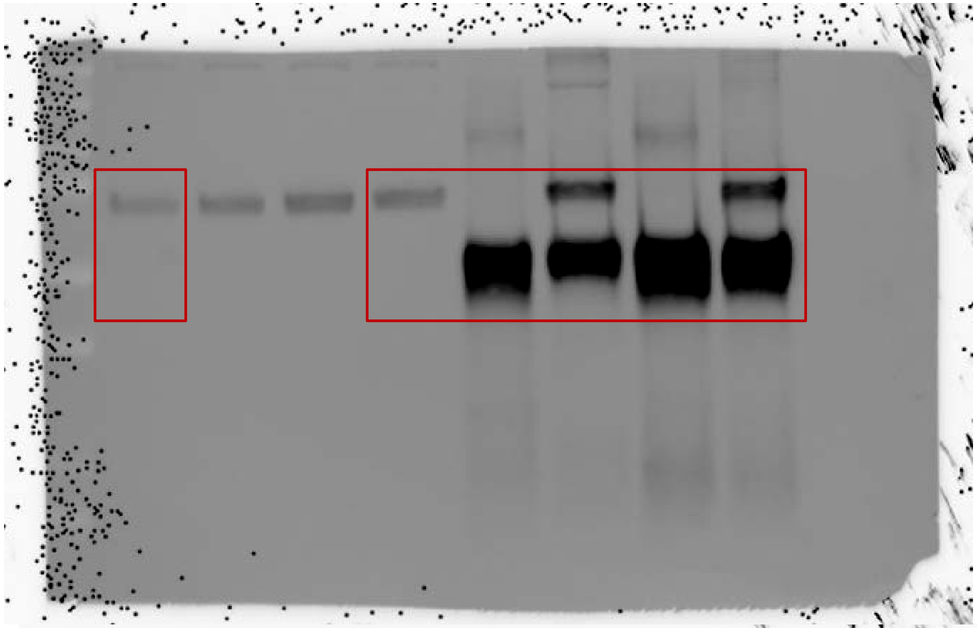

INTS9

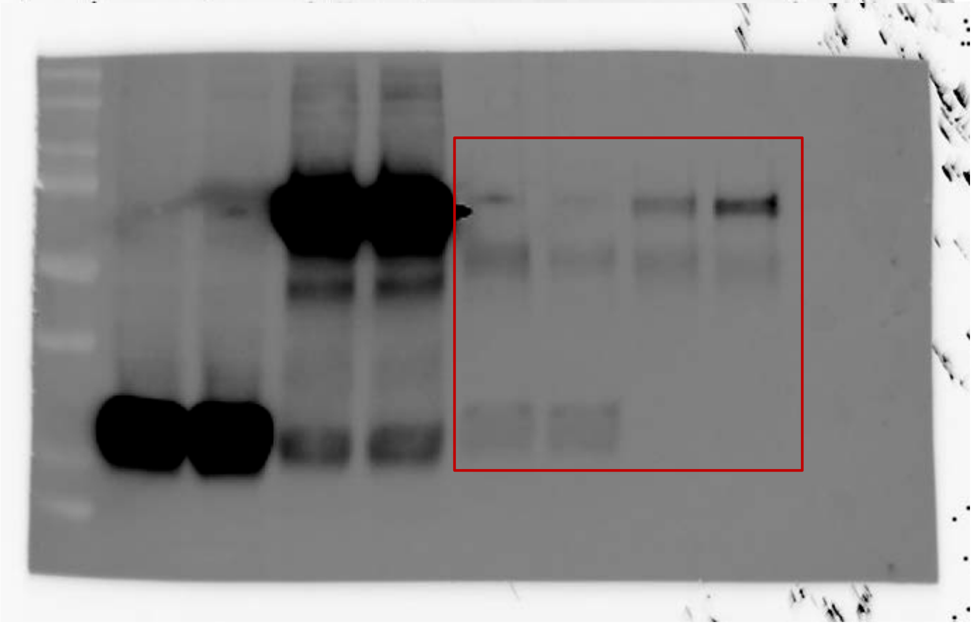

GFP

Uncroped blots corresponding to figure 1E

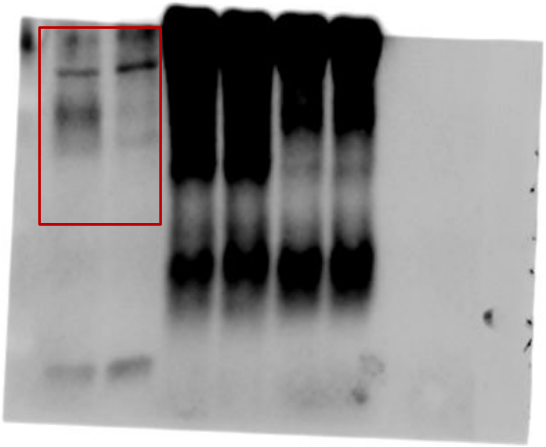

WDR73

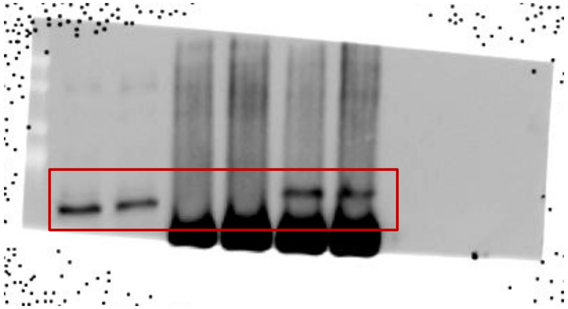

INTS9

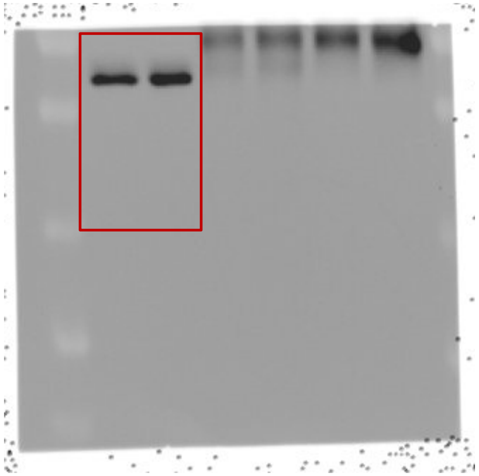

actin

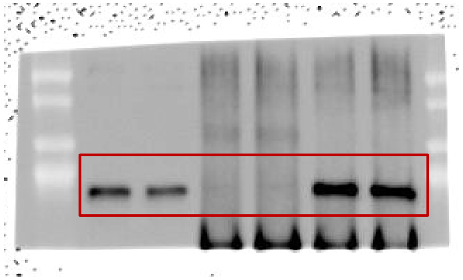

INTS11

Uncroped blots corresponding to figure 1F

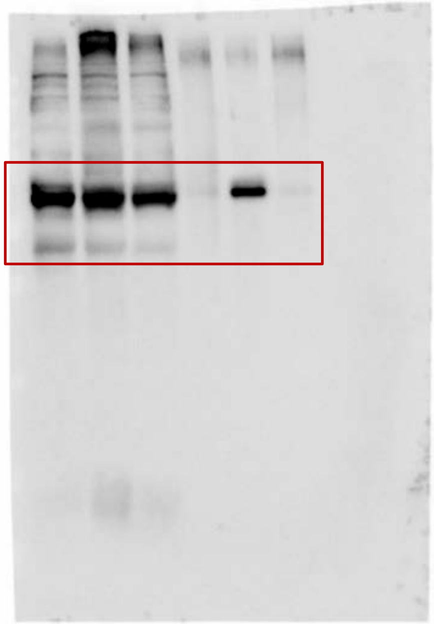

INTS11

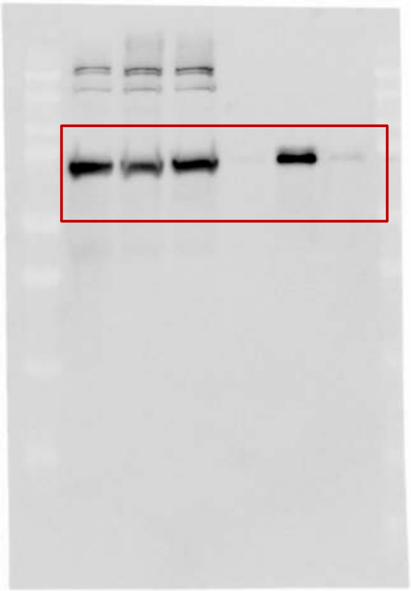

INTS9

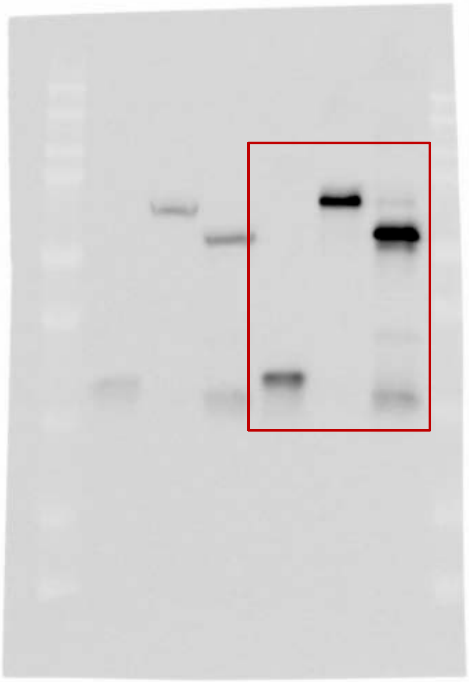

GFP

Uncroped blots corresponding to figure 1G

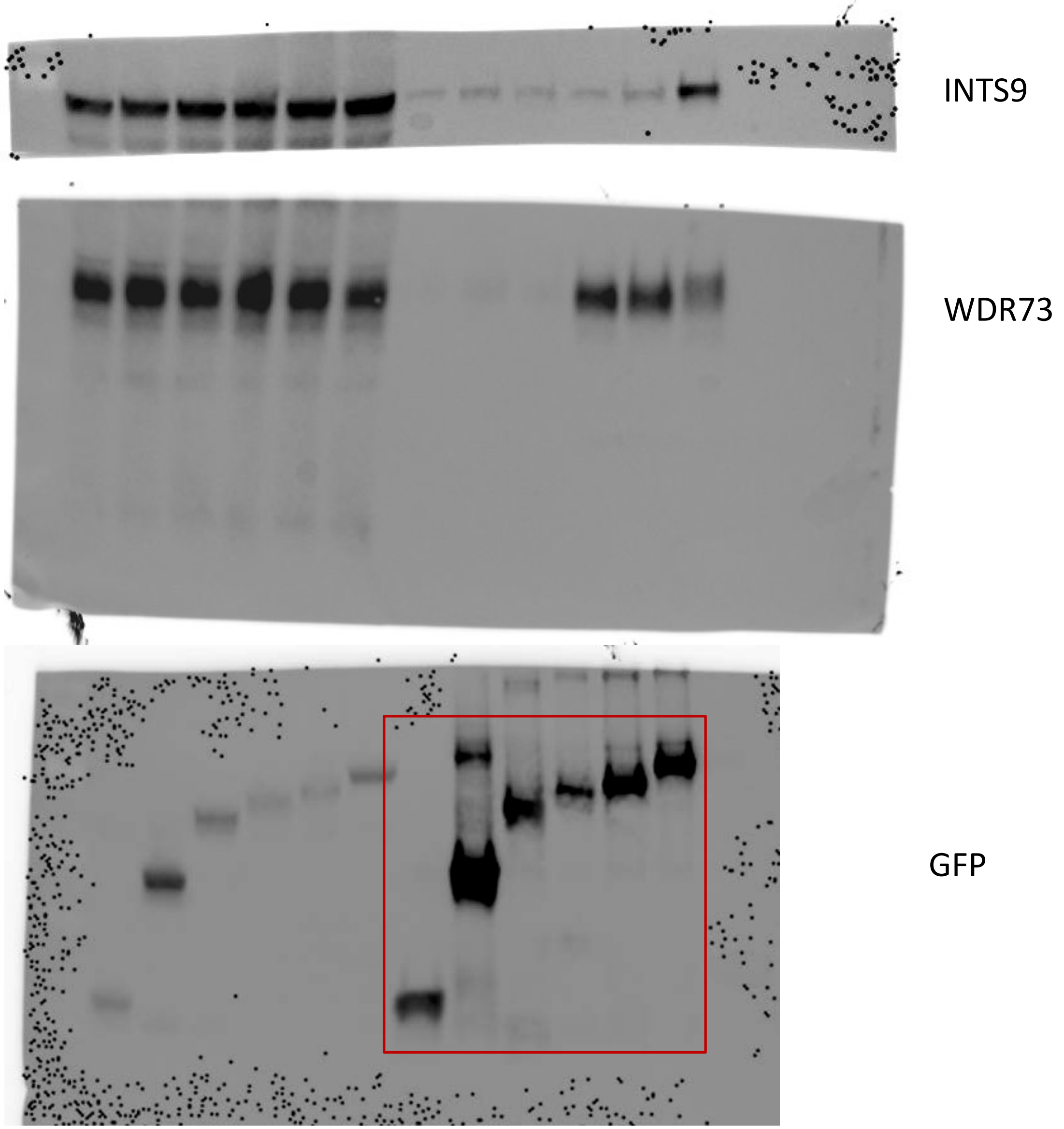

Uncroped blots corresponding to suplementary figure S2

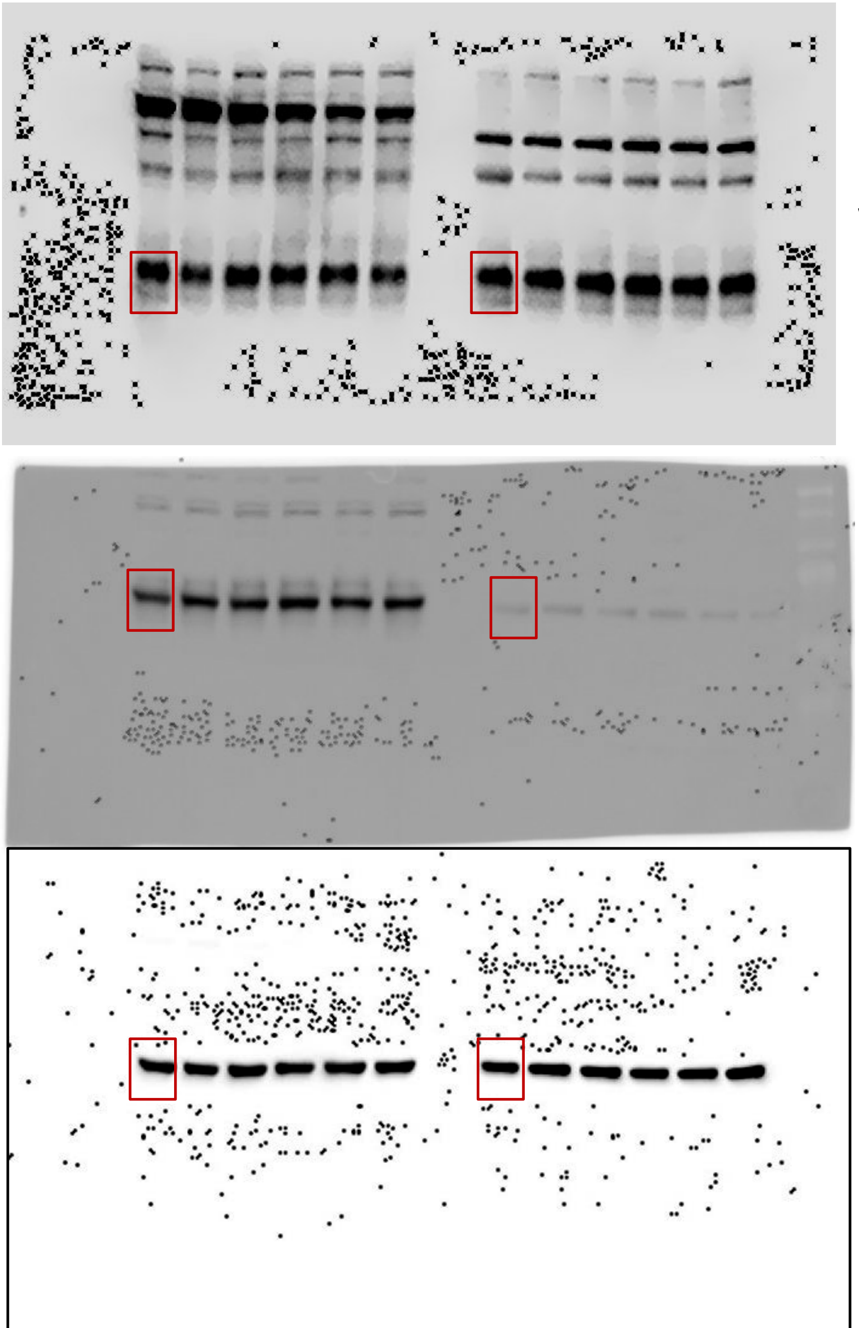

Uncroped blots corresponding to supplementary figure S3A

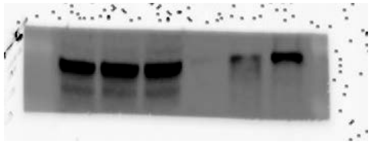

INTS9

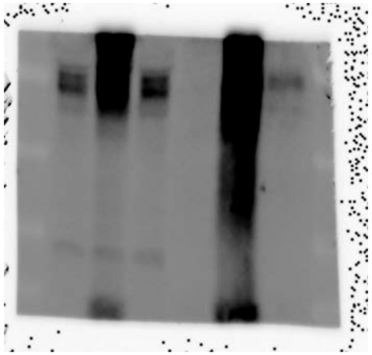

WDR73

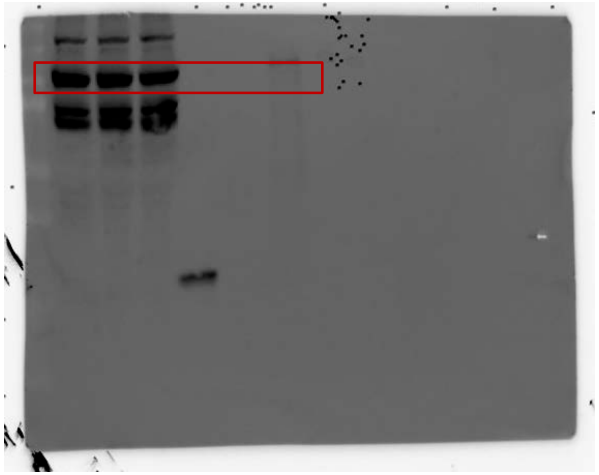

INTS4

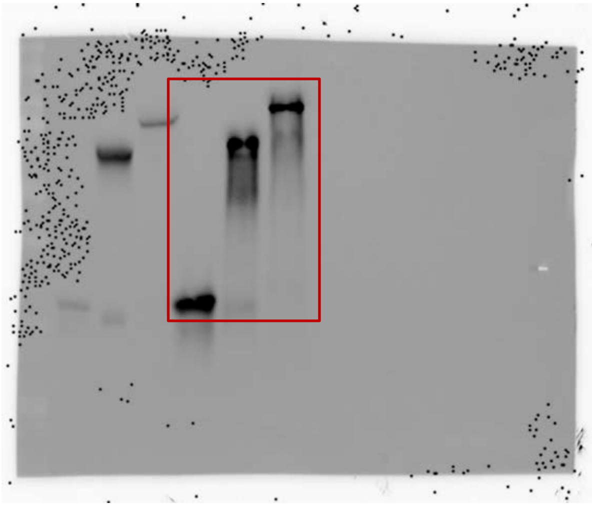

GFP

Uncroped blots corresponding to supplementary figure S3C

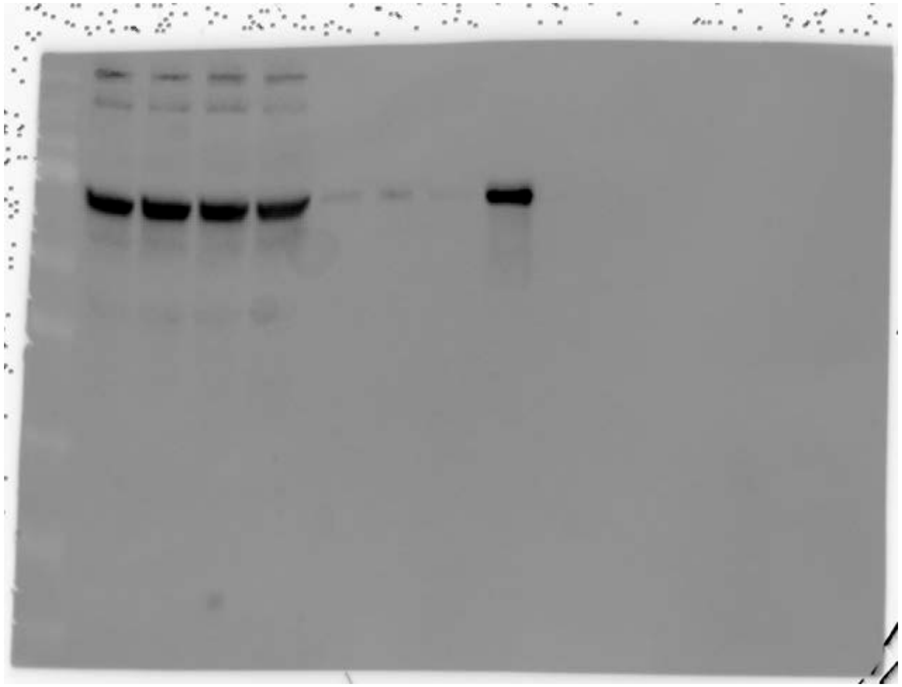

INTS9

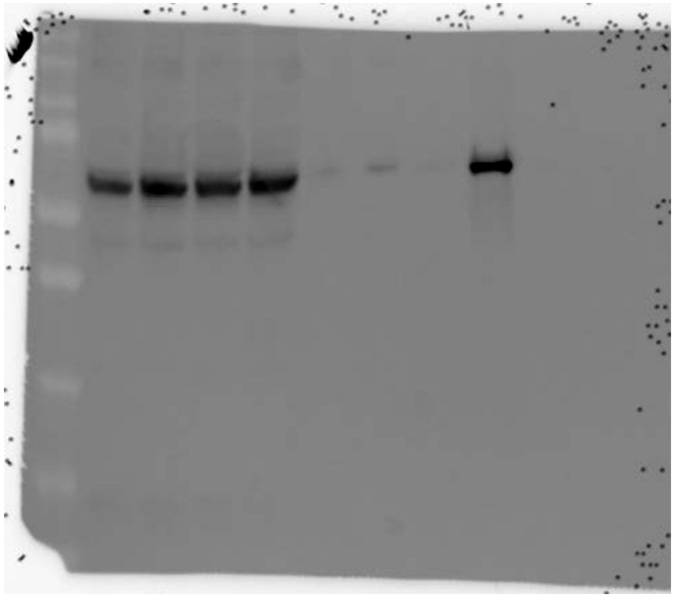

INTS11

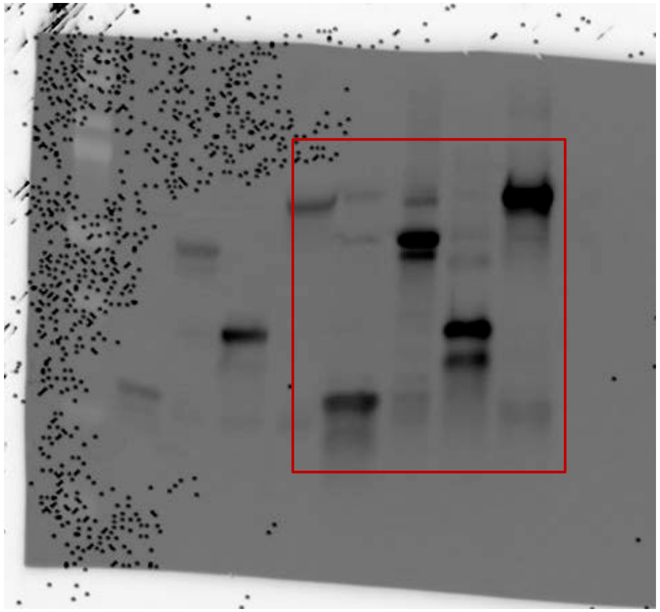

GFP

Uncroped blots corresponding to supplementary figure S3Di

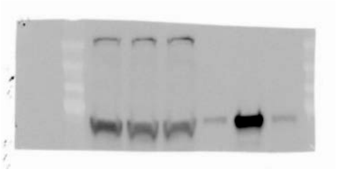

INTS9

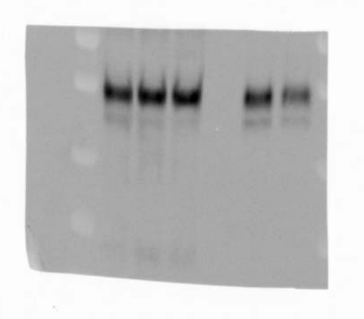

WDR73

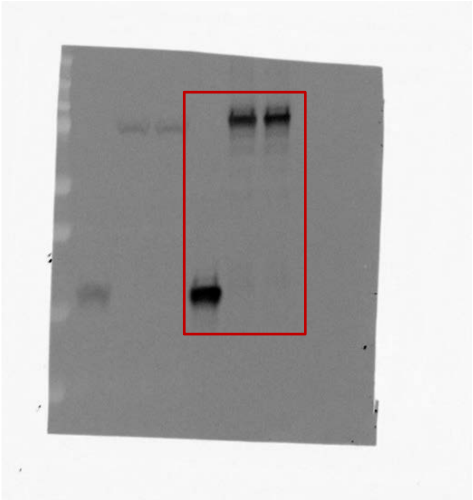

GFP

Uncroped blots corresponding to supplementary figure S5Ai

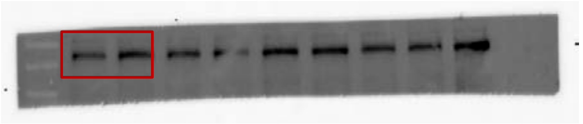

EGFR

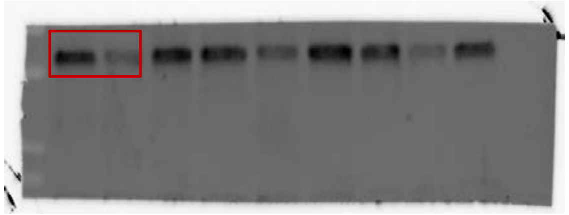

WDR73

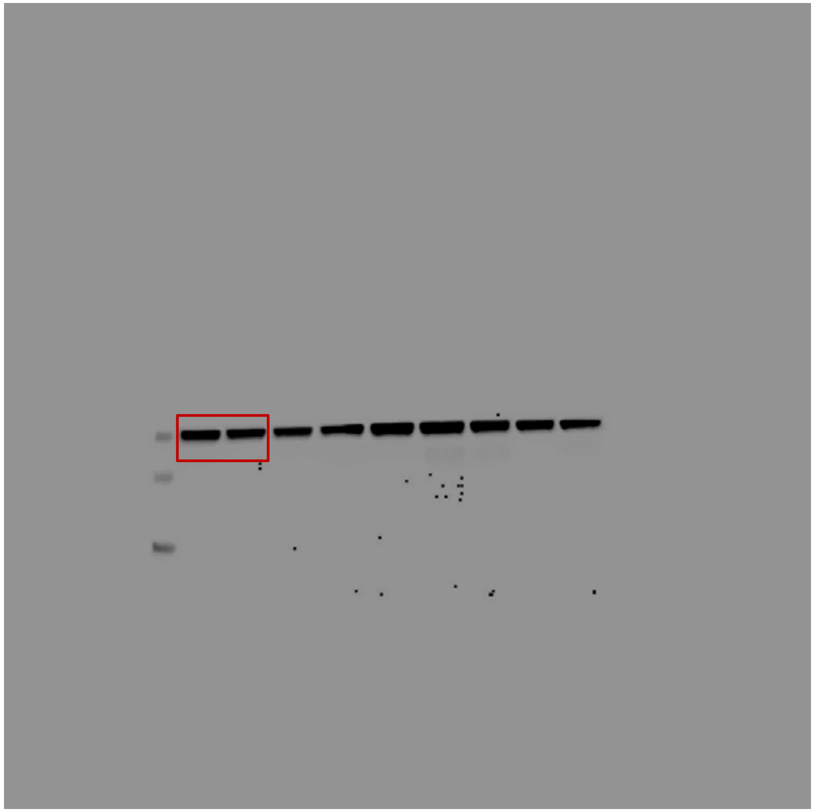

tubulin

Uncroped blots corresponding to supplementary figure S7Ai

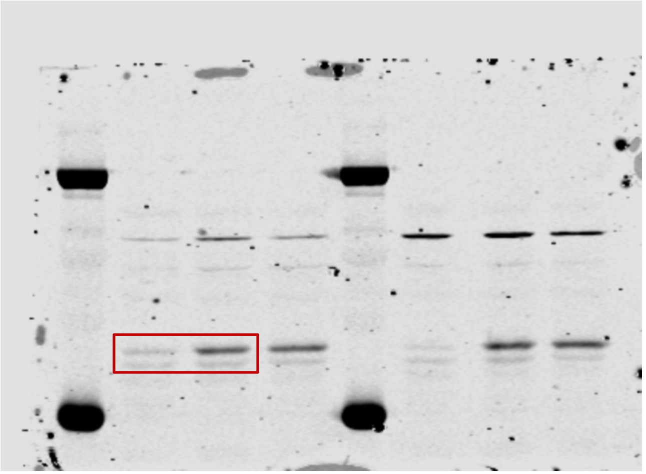

cyclin D1

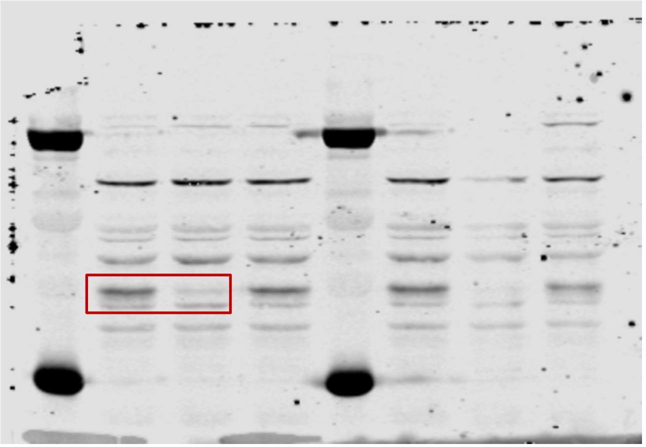

WDR73

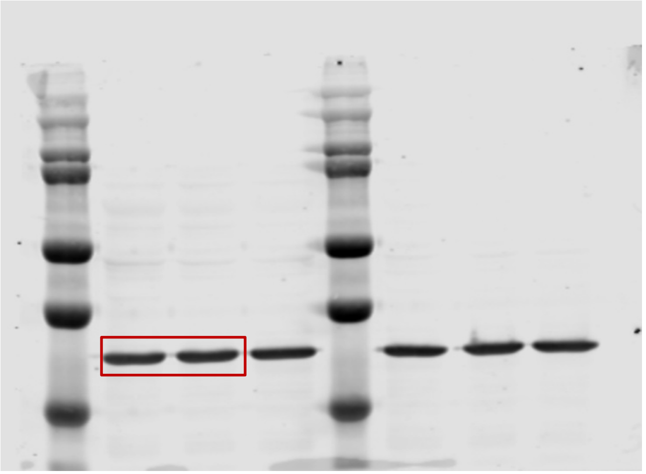

GAPDH

Supplementary Table S8: Primers used for subcloning and site-directed mutagenesis reactions.

**Primer name**	**Sequence 5'-3'**
WDR73 full length NheI forward	GC GCTAGC GATCCTGGGGACGACTGG
WDR73 full length NotI reverse	GCGCGGCCGCTCA GCGGGGGGCACAAAGGTC
WDR73 c.766dupC forward	TTGTCTTCTTGACCCCCCGGGATCTCTGCCATC
WDR73 c.766dupC reverse	GATGGCAGAGATCCCGGGGGGTCAAGAAGACAA
WDR73 1-255 NotI reverse	GCGCGGCCGCTCAGGGGTCAAGAAGACAAAGAC
WDR73 257-378 NheI forward	GCGCTAGCCGGGATCTCTGCCATCCTGTGAG
INTS11 full length NheI forward	GCGCTAGCTGTGGTGCTGGCTTTGGAC
INTS11 full length Not I reverse	GCGCGGCCGCTCAGCTGGGGGCCTGG
INTS9 full length NheI forward	GCGCTAGCAAACTGTATTGCCTGTCAGG
INTS9 length NotI reverse	GCGCGGCCGCTCAGAACTTCTGTAAGAATTTGAGG
INTS11 1-207 Not I reverse	GCGCGGCCGCTCAGGCGTACGTGGACTCTGTG
INTS11 1-390 Not I reverse	GCGCGGCCGCTCAGCTGAATGACATGTACTCCACC
INTS11 1-450 Not I reverse	GCGCGGCCGCTCAGGGGCTTGTGGGCAGC
INTS11 1-493 Not I reverse	GCGCGGCCGCTCACACCAGCCGGAAGTTGC
INTS11 L509A forward	CGCGACCTGCCGCGTGCACCTG
INTS11 L509A reverse	CGCGCCTGGTGCTCAGCCAGACCC

Supplementary Table S9: Primers used in quantitative PCR experiments

Primer name	Sequence 5'-3'
RNU1 (U1) forward	AATGTGGGAAACTCGACTGC *
RNU1 (U1) reverse	TGCAGGCGACATGTTACTTC *
RNU2 (U2) forward	CTTCGGGGAGAGAACAACC *
RNU2 (U2) reverse	GACACTCAAACACGCGTCA *
RNU4 (U4) forward	GCATTGGCAATTTTTGACAG *
RNU4 (U4) reverse	GAACCCCGGACATTCAATC *
RN7SK (7SK) forward	AGGACCGGTCTTCGGTCAA *
RN7SK (7SK) reverse	TCATTTGGATGTGTCTGCAGTCT *
RNU12 (U12) forward	CCTACTTTGCGGGATGCCT
RNU12 (U12) reverse	GACCACCCCACAGTCAGTCTA
SNORD3A forward	CTCTGAACGTGTAGAGCACCG
SNORD3A reverse	CCCGGAGTTTACGAGCTAGTC
FOS forward	CTACCACTCACCCGCAGACT
FOS reverse	AGGTCCGTGCAGAAGTCCT
EGR1 forward	AGCCCTACGAGCACCTGAC
EGR1 reverse	GGTTTGGCTGGGGTAACTG
JUNB forward	ATACACAGCTACGGGATACGG
JUNB reverse	GCTCGGTTTCAGGAGTTTGT
CCND1 (Cyclin D1) forward	ATGTTCGTGGCCTCTAAGATGA
CCND1 (Cyclin D1) reverse	CAGGTTCCACTTGAGCTTGTTC
CCND2 (Cyclin D2) forward	GGACATCCAACCCTACATGC
CCND2 (Cyclin D2) reverse	CGCACTTCTGTTCCTCACAG
CCND3 (Cyclin D3) forward	GCTTACTGGATGCTGGAGGTA
CCND3 (Cyclin D3) reverse	AAGACAGGTAGCGATCCAGGT
CDKN1A (p21) forward	CCTCCCCCTTGTCCTTTC
CDKN1A (p21) reverse	GTGGGACAGGCACCTCAG
HPRT forward	TCTTTGCTGACCTGCTGGATT
HPRT reverse	GTTGAGAGATCATCTCCACCAATTACT
VPS28 forward	TGTCTCCCCCAGCGAGTA
VPS28 reverse	CCTGAAGGCAGCTTTGTATTG
WDR73 forward	ATGGATCCTGGGGACGAC
WDR73 reverse	AGCCAGCAACAAAGACTCC
INTS11 forward	CACCTCCCAGATGATCAAAGA
INTS11 reverse	GCCTGCATAGTAGGCCTTGA

* As described in Oegema *et al.* (2017).

Supplementary Methods:

Generation and quality control of neural progenitor cells (NPCs)

Peripheral blood monocytes (PBMCs) were reprogrammed in induced pluripotent stem cells (iPSCs) at the *Imagine* Institute iPSC facility using non-integrating Sendaï virus approach (CytoTune IPS 2.0 Sendaï Reprogramming kit, THERMO FISHER SCIENTIFIC, lot 21300018) in the reprogramming medium StemPro-34 medium (THERMO FISHER SCIENTIFIC) supplemented with IL-3, IL-6, SCF, Flt3-L, then StemPro-34 without cytokines on a vitronectin reprogramming support.

To obtain neural progenitor cell lines (NPCs), iPSCs were differentiated using a dual SMAD inhibition strategy (Feyeux *et al.*, 2012). To ensure the correct identity of the NPCs used in this study, we performed a flow-cytometry based quality control check. Cells were first isolated using trypsin, filtered on a 40µm strainer and counted to have an equal number of cells for each cell line tested and all controls. Trypsin was then inactivated by adding an equal volume of Opti-MEM (GIBCO) 10% FCS and then centrifuged for 5min at 1,200rpm at 4°C before resuspension in Fc blocking solution [Fc blocking reagent diluted 1/5 in FACS buffer (PBS, 2.5% FCS, 2.5mM EDTA)]. Cells were incubated for 15 min on ice before washing in FACS buffer and a second centrifugation at 1,200rpm for 5 min at 4°C. The pellets were resuspended in fluorescently-conjugated primary antibodies CD57-FITC (HNK1) and CD271-PE (P75) diluted 1/10 in FACS buffer and incubated on ice for 30min. The cells were then again washed in FACS buffer, centrifuged a final time at 1,200rpm for 5min at 4°C before resuspension in FACS buffer. Cells were then acquired on LSR Fortessa flow cytometer (BD). Cells passed the quality control check if the population of $HNK1^+/P75^-$ cells was greater than 80%.

The NPC controls were derived from the iPSC lines, IMAGINi005 (clone #04) and IMAGINi009 (clone #09), and are referred to as Ctrl 05-04 and Ctrl 09-09 in this study.

Nucleo-cytoplasmic fractionation

To perform a nucleo-cytoplasmic fractionation, cells were first lysed in a low salt buffer (10mM Tris-HCl pH7.8, 10mM KCl, 1.5mM $MgCl_2$, 0.5mM DTT plus complete protease inhibitor). Following lysis, cells were incubated on ice for 10min, followed by centrifugation at 13,500rpm for 10min at 4°C. The supernatant, containing the cytoplasmic extract, was removed at this point for subsequent analysis. The pellet, containing the nuclear proteins, was washed once in low salt buffer and again centrifuged for 10min at 4°C. The pellet was then resuspended in 100µl of high salt buffer (20mM Tris-HCl pH7.9, 420mM KCl, 1.5mM $MgCl_2$, 10% glycerol, 0.5mM DTT plus complete protease inhibitor) and allowed to rotate overnight at 4°C. The following day, the lysate was again centrifuged at 13,000rpm for 10min, and the supernatant, consisting of the nuclear extract, removed for analysis.

Proteomic analysis

Proteomic analysis was carried out by the Proteomic Platform 3P5-Necker (Necker Hospital, Paris, France).

- **NanoLC-MS/MS protein identification and quantification**

S-Trap micro spin column (PROTIFI, Hutington, USA) digestion was performed on IP eluates according to manufacturer's protocol. Briefly, samples were digested with 3µg of trypsin (PROMEGA) at 37°C overnight. After elution, peptides were vacuum dried.

Samples were resuspended in 35 µL of 10% acetonitrile, 0.1% trifluoroacetic acid in high performance liquid chromatography (HPLC)-grade water. For each run, 5 µL was injected in a nanoRSLC-Q Exactive PLUS (RSLC Ultimate 3000) (THERMO SCIENTIFIC, Waltham MA, USA). Peptides were loaded onto a µ-precolumn (Acclaim PepMap 100 C18, cartridge, 300 µm i.d.×5 mm, 5 µm) (THERMO SCIENTIFIC), and were separated on a 50 cm reversed-phase liquid chromatographic column (0.075 mm ID, Acclaim PepMap 100, C18, 2 µm) (THERMO SCIENTIFIC). Chromatography solvents were (A) 0.1% formic acid in water, and (B) 80% acetonitrile, 0.08% formic acid. Peptides were eluted from the column with the following gradient 5% to 40% B (38 min), 40% to 80% (1 minute). At 39 min, the gradient stayed at 80% for 4 min and, at 44 min, it returned to 5% to re-equilibrate the column for 16 min before the next injection. One blank was run between each series to prevent sample cross-contamination. Peptides eluted from the column were analyzed by data dependent MS/MS, using top-10 acquisition method. Peptides were fragmented using higher-energy collisional dissociation (HCD). Briefly, the instrument settings were as follows: resolution was set to 70,000 for MS scans and 17,500 for the data dependent MS/MS scans in order to increase speed. The MS automatic gain control (AGC) target was set to 3.10^6 counts with maximum injection time set to 200 ms, while MS/MS AGC target was set to 1.10^5 with maximum injection time set to 120 ms. The MS scan range was from 400 to 2000 m/z. Dynamic exclusion was set to 30 seconds duration.

- **Data Processing Following LC-MS/MS acquisition**

The MS files were processed with the MaxQuant software version 1.5.8.3 and searched with Andromeda search engine against the database of *Homo Sapiens* from swissprot 07/2017. To search parent mass and fragment ions, we set an initial mass deviation of 4.5 ppm and 20 ppm respectively. The minimum peptide length was set to 7 amino acids and strict specificity for trypsin cleavage was required, allowing up to two missed cleavage sites. Carbamidomethylation (Cys) was set as fixed modification, whereas oxidation (Met) and N-term acetylation were set as variable modifications. Match between runs was not allowed. Label-free quantification (LFQ) minimum ratio count was set to 1. The false discovery rates (FDRs) at the protein and peptide level were set to 1%. Scores were calculated in MaxQuant as described previously (Cox et al., 2008). The reverse and common contaminants hits were removed from MaxQuant output. Proteins were quantified according to the MaxQuant label-free algorithm using LFQ intensities (Cox et al., 2014; Luber et al., 2010)

Dara were analyzed with Perseus software (version 1.6.0.7) freely available at www.perseus-framework.org (Tyanova et al., 2016). The LFQ data were transformed in log2. All the proteins identified in at least 5 of the 6 biological replicates per group were submitted to statistical test (volcano plot, FDR=0.05 and S0=2) after imputation of the missing value by a Gaussian distribution of random numbers with a standard deviation of 30% relative to the standard deviation of the measured values and 3 standard deviation downshift of the mean to simulate the distribution of low signal values. Protein annotations (GO, Keywords) were retrieved directly using via Perseus.

Flow cytometry

For flow cytometry analysis, an equal number of cells per condition and all controls were fixed in 2% PFA for 30 min on ice. Cells were washed twice in cold PBS, then resuspended in 3ml ice-cold 70% ethanol and kept for at least 24hrs overnight before further processing. The following day, cells were centrifuged at 1,500rpm for 5min at 4°C, washed twice in PBS before resuspension in a solution containing primary

antibody diluted to the required concentration in 100µl PBS 0.5% Triton X-100, 1% BSA. Cells were stained for 30min on ice, then washed and resuspended in an Alexa-488 conjugated secondary antibody (LIFE TECHNOLOGIES) diluted 1/400 in PBS 0.5% Triton X-100, 1% BSA. After a 30minute incubation, cells were again washed in PBS, centrifuged for 5min at 1,500rpm and then resuspended in propidium iodide (PI) staining solution (PBS 0,5% TX-100, 2mg RNAse A and 250mg PI). Cells were incubated at room temperature for 30min with minimal exposure to light before acquisition on the flow cytometer (Kaluza for Gallios, BECKMAN COULTER). Analysis was performed using the Kaluza analysis software (BECKMAN COULTER).

Immunofluorescence

For immunofluorescence experiments, NPCs were seeded onto glass-coverslips precoated with Poly-$_{L}$-Ornithine and laminin at a minimum density of $1x10^6$ cells/well of a 6-well dish. 24-48hrs hours after seeding, cells were fixed in 4% paraformaldehyde (PFA) diluted in PBS. After 20min, the PFA was removed and cells washed once in PBS before a 5minute incubation in 30mM glycine (SIGMA) to quench the PFA autofluorescence. Cells were then washed a further 2x in PBS and then permeablised by incubation in 0.5% Triton X-100 diluted in PBS for 5min. Cells were washed 3x in PBS and then incubated in PBS 1% BSA overnight at 4°C. The following day, coverslips were incubated in primary antibody diluted to the appropriate concentration in PBS 1% BSA, and then washed 3x before incubation in an Alexa-488 conjugated secondary antibody (LIFE TECHNOLOGIES) diluted 1/400, also in PBS 1% BSA. The coverslips were then washed a final 2x in PBS, once in water, and finally mounted onto glass slides using Mowiol mounting solution. Images were captured using a LEICA TCS SP8 SMD (Single Molecule Detection) confocal microscope.

Design of qPCR primers for U12 and SNORD3A

In order to design primers that amplified the long, unprocessed *U12* transcript, we first retrieved the mature U12 RNA sequence, plus 200 nucleotides downstream of the 3' end, from BioMart. We cross-checked this sequence with that available on NCBI gene. As the sequence of the 3' box in U12 has been previously described (Tarn et al., 1995), we placed the reverse primer downstream of this region. In regards to *SNORD3A,* our goal was to design primers that amplified a long and in theory, unprocessed, transcript. To do this, we used BioMart to retrieve the sequence encoding SNORD3A plus 200 nucleotides downstream of the 3' end. We placed the forward primer in the sequence of the mature *SNORD3A* transcript, and the reverse primer in the downstream sequence. Using the U1 3'box consensus sequence and the motif matching FIMO software, we were unable to locate a putative 3'box in this region, but as reports state that 3' cleavage occurs between 9-19 nucleotides downstream of the final nucleotide in the mature sequence (Hernandez, 1985), we ensured the reverse primer was beyond this region.

RNA-Sequencing and subsequent analysis

RNA-sequencing was performed on RNA extracted from human immortalised podocytes in which WDR73 was depleted using a commercially available siRNA pool, with podocytes treated with a non-targeting oligonucleotide used as a control. 48hrs following siRNA transfection, cells were subjected to 24hrs starvation in complete media supplemented with 0.1% FCS before stimulation with 100ng/ml EGF for 30min. The experiment was performed in quadruplicate, with the three samples in which depletion of WDR73, as determined by RT-qPCR, was most comparable being those submitted to the genomic platform of Imagine Institute for subsequent analysis.

RNA-Seq libraries were prepared using 1µg of total RNA using the Universal Plus mRNA-Seq kit (NUGEN) as recommended by the manufacturer. Briefly, mRNA was captured using polyA+ magnetic beads. The mRNA were then fragmented chemically. Single strand and second strand cDNA were produced and then ligated to Illumina compatible adapters with Unique Dual Index. Following an initial test to evaluate the suitable number of PCR cycles to apply to each sample, the cDNA produced were amplified by PCR. To produce oriented or 'stranded' RNA-Seq libraries, a final strand selection was performed. An equimolar pool of the final indexed RNA-Seq libraries was prepared (the NuQuant system from NUGEN was used to facilitate the RNAseq libraries quantification and normalization) and sequenced on a NovaSeq6000 from Illumina (Paired-End reads 100 bases + 100 bases). A total of ~50 millions of passing filter paired-end reads was produced per library.

FASTQ files were then mapped to the ENSEMBL Human (GRCh38/hg38) reference using Hisat2 and counted by featureCounts from the Subread R package (R version 3.5.2; 2018-12-20). Read count normalisations and groups comparisons were performed by three independent and complementary statistical methods: Deseq2 (version 1.20), edgeR (version 3.22.5), LimmaVoom (version 3.36.5). Flags were computed from counts normalized to the mean coverage. All normalized counts <20 were considered as background (flag 0) and >=20 as signal (flag=1). P50 lists used for the statistical analysis regroup the genes showing flag=1 for at least half of the compared samples. The results of the three methods were filtered at pvalue<=0.05 and folds 1.2/1.5/2 compared and grouped by Venn diagram. Functional analyses were carried out using Ingenuity Pathway Analysis (IPA, QIAGEN).
